# Antiproliferative activity and p53 upregulation effects of chalcones on human breast cancer cells

**DOI:** 10.1080/14756366.2019.1615485

**Published:** 2019-05-23

**Authors:** Mariana Bastos dos Santos, Daiane Bertholin Anselmo, Jéssica Gisleine de Oliveira, Bruna V. Jardim-Perassi, Diego Alves Monteiro, Gabriel Silva, Eleni Gomes, Ana Lucia Fachin, Mozart Marins, Débora Aparecida Pires de Campos Zuccari, Luis Octavio Regasini

**Affiliations:** a Department of Chemistry and Environmental Chemistry, Institute of Biosciences, Humanities and Exact Sciences (IBILCE), São Paulo State University (UNESP), São Paulo, Brazil;; b Department of Molecular Biology, Medicine College of São José do Rio Preto (FAMERP), São Paulo, Brazil;; c Department of Biology, Institute of Biosciences, Humanities and Exact Sciences (IBILCE), São Paulo State University (UNESP), São Paulo, Brazil;; d Biotechnology Unit, University of Ribeirão Preto (UNAERP), São Paulo, Brazil

**Keywords:** Chalcones, cancer, p53, antiproliferative, apoptosis

## Abstract

Chalcones are valuable structures for drug discovery due to their broad bioactivity spectrum. In this study, we evaluated 20 synthetic chalcones against estrogen-receptor-positive breast cancer cells (MCF-7 line) and triple-negative breast cancer (TNBC) cells (MDA-MB-231 line). Antiproliferative screening by MTT assay resulted in two most active compounds: 2-fluoro-4’-aminochalcone (**11**) and 3-pyridyl-4’-aminochalcone (**17**). Their IC_50_ values ranged from 13.2 to 34.7 µM against both cell lines. Selected chalcones are weak basic compounds and maintained their antiproliferative activity under acidosis conditions (pH 6.7), indicating their resistance to ion-trapping effect. The mode of breast cancer cells death was investigated and chalcones **11** and **17** were able to induce apoptosis rather than necrosis in both lines. Antiproliferative target investigations with MCF-7 cells suggested **11** and **17** upregulated p53 protein expression and did not affect Sp1 protein expression. Future studies on chalcones **11** and **17** can define their *in vivo* therapeutic potential.

## Introduction

Breast cancer is the most common type of cancer that affects women around the world, corresponding to 25% of cases. It is also the main cause of cancer death among women^1–3^. Its classification is based on the presence of cellular receptors: (i) Hormone-Receptor (HR), with Estrogen (ER) and/or Progesterone-Receptors (PR); (ii) Human Epidermal Growth Factor-2 receptor (HER2); and (iii) Triple-Negative Breast Cancer (TNBC), which does not express ER, PR, or HER2 receptors^4–6^. Chemotherapy choices for breast cancer are made according to its classification aiming to reach specific targets. ER-positive cancer treatments include ER modulators, such as tamoxifen or aromatase inhibitors. HER2-positive cancer is treated with monoclonal antibodies, such as trastuzumab, which is administered alongside tyrosine-kinase inhibitors^7–9^. TNBC cancer treatment is focused on cytotoxic agents, such as taxanes or doxorubicin. This cancer has poor response to chemotherapy, particularly at metastatic sites, with survival rates below 2 years. Altogether, TNBC cancer has been considered the most severe and difficult to treat due to a lack of targeted therapy^10–12^.

Chalcones are valuable structures for drug discovery due to their broad bioactivity spectrum, as well as their versatile and simple synthesis. Their structures, bearing two benzene rings (A and B), are linked by an enone bridge. They have demonstrated antiproliferative activity against cancer cells, including breast cancer^13–15^. Mai et al. described chalcone was effective against ER-positive breast cancer cells (MCF-7 line) targeting 20 apoptotic markers^16^. Iftikhar et al. reported that chalcone bearing chlorine at position 2 was effective against breast cancer cells (CAL-51 line) and induced accumulation of p53 protein^17^. Silva and coauthors described unsubstituted chalcone increased p53 protein activity in osteosarcoma cells (U2OS line) through the induction of heat shock protein DNAJB1^18^.

In our ongoing search for anticancer compounds with structures based on chalcone framework, we evaluated the antiproliferative activity of 20 chalcones against ER-positive cells (MCF-7 line) and TNBC (MDA-MB-231 line). The two most active chalcones (**11** and **17**) were selected to antiproliferative evaluation under acidosis conditions (pH 6.7) and pro-apoptotic activity in MCF-7 and MDA-MB-231 lines. In addition, we investigated tumour molecular targets of **11** and **17** in MCF-7 line, which were able to upregulate p53 protein expression.

## Materials and methods

### Chemical procedure for synthesis of chalcones 1–20

Reagents and solvents were purchased from Merck^®^ (Kenilworth, NJ). Series of 20 chalcones was synthesised by Claisen–Schmidt aldol condensation reaction, according to protocol described by Santos and coauthors, with minor modifications^19^^,^^20^. Reactions were carried out at room temperature using 3.0 mmol of 4'-aminoacetophenone and 3.0 mmol of aldehydes, which were dissolved in ethanol (30 mL). Sodium hydroxide in ethanol (1.0 mol/L) was added as catalyst solution. Reagents conversion was monitored using thin layer chromatography. Crude product was poured onto ice (from distilled and deionised water) and filtered. All compounds were purified over silica gel chromatography column eluted with mixture of hexane and ethyl acetate (3:2). Melting points were determined in TecnoponPFM-II^®^ apparatus (MS Tecnopon Instrumentação, Piracicaba, Brazil) and were uncorrected. Structure of compounds was confirmed by ^1^H and ^13^C nuclear magnetic resonance (NMR) spectra analyses. Spectral data were obtained in Bruker Avance III^®^ (14 Tesla, 600 MHz) equipment (Bruker Corporation, Billerica, MA) using deuterated dimethyl sulfoxide (DMSO-d_6_) as solvent. Chalcones had their UV–vis spectra and chromatograms obtained in High Performance Liquid Chromatography with Diode Array Detector (HPLC-DAD) Agilent Technologies^®^ 1220 Infinity equipment (Agilent Technologies, Palo Alto, CA) coupled with a photodiode array system (1260-Infinity^®^) and Agilent Zorbax Eclipse Plus C-18^®^ column (250 mm × 4.6 mm, 5 μm), using methanol:water (3:1) as mobile phase (1.0 mL/min).

### Antiproliferative activity of chalcones 1–20

Human breast cancer cell lines MCF-7 (HTB-22) and MDA-MB-231 (HTB-26) were purchased from American Type Culture Collection (ATCC). Both cell lines were cultured in DMEM (Gibco^®^, Carlsbad, CA) supplemented with 10% fetal bovine serum (FBS-LGC^®^), penicillin-streptomycin (100 μg/mL, Merck^®^, Kenilworth, NJ). Both cell lines were incubated at 37 °C under humidified atmosphere with 5% CO_2_ (Thermo Fischer Scientific^®^ Incubator, Waltham, MA).

Antiproliferative activity of chalcones **1**–**20** was evaluated by MTT assay^21–23^. Cells were seeded in 96-well plate, with an initial cell density of 1 × 10^4^ and 2.5 × 10^4^ cells/well of MCF-7 and MDA-MB-23, respectively. Cells were cultured with DMEM supplemented with 10% FBS and compounds at 20 μM for 48 h at 37 °C under 5% CO_2_ humidified atmosphere. Cells were incubated with MTT (Sigma Aldrich^®^, St. Louis, MO) solution (1 mg/mL) for 40 min. Formazan crystals were solubilised in dimethylsulfoxide (DMSO, Sigma Aldrich^®^, St. Louis, MO), and absorbance rates were measured at 562 nm in ThermoPlate^®^ TP Reader. Chalcones **1**, **6**, **8**, **9**, **11**, **17**, **19**, and **20** had the highest antiproliferative activity and were assayed against both cell lines at seven concentrations, ranging from 1.25 to 80 μM for IC_50_ values determination.

### Antiproliferative activity of selected chalcones 11 and 17 under acidosis

Cell viability was evaluated under acidosis conditions by MTT assay, using 4-morpholine-ethanesulfonic acid to produce a pH value of 6.7^24^^,^^25^. Cells were treated with selected chalcones **11** and **17** at their respective IC_50_ values. All procedures were performed in triplicate and three independent experiments.

### Pro-apoptotic activity of selected chalcones 11 and 17

Pro-apoptotic activity of selected chalcones **11** and **17** was evaluated using FITC Annexin V Apoptosis Detection Kit I (BD Pharmingen^®^, BD Biosciences, Franklin Lakes, NJ) by flow cytometry^26^. MCF-7 and MDA-MB-231 cells were seeded and cultivated in 6-well plates (6.5 × 10^5^ cells/well), for 24 h, and treated with 40 μM selected chalcones in DMEM containing 10% FBS for 48 h. As negative control 0.1% DMSO was used. Adherent and floating cells were collected, submitted to centrifugation, and washed with PBS (Sigma Aldrich^®^, St. Louis, MO). Apoptotic cells were determined by flow cytometry in BD FACSCalibur^®^ Flow Cytometer (BD Biosciences^®^ Franklin Lakes, NJ), after double staining (Annexin V/propidium iodide) using FITC Annexin V Apoptosis Detection kit I (BD Pharmigen^®^, BD Biosciences, Franklin Lakes, NJ) according to instructions of the manufacturer. All procedures were performed in triplicate and in three independent experiments.

### Effect of selected chalcones 11 and 17 on Sp1 and p53 proteins expressions

Protein expression was assessed using western blot assay. Breast cancer cells (MCF-7) were seeded in 6-well plates at 7.5 × 10^5^ cells/well, treated with selected chalcones **11** and **17** at 10 and 20 μM or with negative control (0.1% DMSO) for 24 h^26^. Cells were suspended in PBS solution, collected in Radioimmunoprecipitation Assay buffer – (RIPA buffer at 4 °C), supplemented with proteinase inhibitors, sonicated for lysis, and submitted to centrifugation (14,000 rpm, 4 °C, 20 min, Eppendorf^®^, Hamburg, Germany). Protein total concentration was obtained using Pierce™ BCA Protein Assay reagent (Thermo Scientific^®^, Waltham, MA). Total proteins (30 μg) in sodium dodecyl sulphate (SDS) buffer were warmed at 95 °C, cooled at 4 °C and submitted to sodium dodecyl sulphate–polyacrylamide gel electrophoresis SDS-PAGE (12%, 100 V, 360 mA) for 90 min at room temperature, and were transferred to nitrocellulose membrane (PALL Corporation^®^, Port Washington, NY). Membranes were blocked using TBST buffer (25 mM Tris, 3 mM KCl, 0.14 M NaCl, 0.05% Tween 20) containing 5% nonfat milk at room temperature for 1 h, followed by overnight incubation at 4 °C, with primary antibodies to Sp1 (MilliPore^®^, Burlington, MA), p53, and β-actin (Santa Cruz Biotechnology^®^, Dallas, TX). Subsequently, membranes were washed with TBST (3×) and incubated with peroxidase-conjugated secondary antibodies diluted in TBST buffer-5% nonfat milk (1:5000) at room temperature and washed several times with TBST buffer. Proteins were detected through chemiluminescence using Enhanced Chemiluminescent (ECL^®^) western blotting detection reagent (Amersham Biosciences^®^, Little Chalfont, UK) in LAS 500 luminescence analyser (GE Healthcare^®^, Chicago, IL)^26^. Bands intensity in image was quantified using ImageJ^®^ software (NIH Image J system, Bethesda, MD).

### Statistical analysis

Statistical analyses were carried out using one-way ANOVA multiple comparison tests followed by Tukey’s HSD test^19^^,^^27^. Statistical significance difference was considered if *p*< 0.05.

## Results and discussion

### Chemistry

**Scheme 1.**
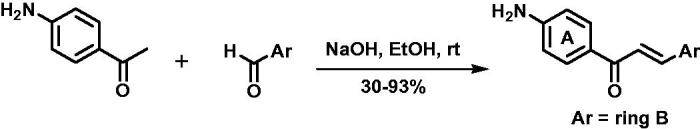
Claisen-Schmidt reaction for synthesis of chalcones **1**–**20.**

Chalcones **1**–**20** were synthesised by Claisen–Schmidt reaction with yields of 30–93% after chromatography purification. Series of compounds has chalcones with amino at position 4’ (ring A) and benzene ring B substituted by electron-withdrawing (EWG) and electron-donating groups (EDG). Also, aryl analogues with benzene ring B replaced for furan, thiophene, pyridine, and naphthyl rings were synthesised (Scheme 1).

All NMR parameters, including hydrogen and carbon chemical shifts (*δ*_H_ and *δ*_C_ in ppm), integrations, multiplicities, and coupling constants (*J* in Hz), corresponded to proposed structures of chalcones **1**–**20**. Two main signals in ^1^H NMR spectra were diagnosed: (i) a pair of doublets with *J* ranging from 15 to 16 Hz (7.43–8.10 ppm), attributed to methyne hydrogens of *trans* carbon–carbon double bonds and (ii) broad singlets between 6.18 and 6.27 ppm related to geminal hydrogens of amino group. In ^13^C NMR spectra, signals in 185.9–186.1 confirmed ketone carbonyls, which are shifted to downfield due to conjugation with carbon–carbon double bonds. UV–Vis had *λ*_max_ at 298–378 nm, which were related to a pivotal chromophore of chalcone framework (conjugation between ring B and enone bridge). HPLC-DAD analyses of peak areas integrations indicated chalcones purity of 94.0–99.9%. All chromatograms and spectra are presented in supplemental data (Figures S1–S62).

### Antiproliferative activity of chalcones 1–20

Preliminary antiproliferative activity of chalcones **1**–**20** was evaluated by MTT assay for 48 h against MCF-7 (ER) and MDA-MB-231 (TNBC) lines. Percentages of metabolically viable cells (%MVC) are summarised in Table 1. Series of compounds demonstrated active (e.g. **20**) and inactive (e.g. **3**) chalcones, suggesting molecular variations were relevant for antiproliferative screening against both cell lines. Experiments at 20 μM allowed us to derive preliminary structural features related to antiproliferative activity^28–30^.

**Table 1.** Percentage of metabolically viable cells (%MVC) treated with chalcones **1**–**20** (at 20 μM) and IC_50_ values (in μM) of selected chalcones.


Cpd.	Ar	MCF-7 (ER)		MDA-MB-231 (TNBC)	
		%MVC	IC_50_	%MVC	IC_50_
**1**	Phenyl	97.3 ± 6.0	>100	82.6 ± 4.0	60.3 ± 2.9^c^
**2**	4-Nitrophenyl	97.3 ± 4.9	nd	104.1 ± 3.8	nd
**3**	4-Trifluoromethylphenyl	98.3 ± 6.6	nd	100.7 ± 6.7	nd
**4**	4-Cyanophenyl	98.2 ± 2.1	nd	99.7 ± 6.3	nd
**5**	4-Fluorophenyl	93.3 ± 5.7	nd	92.8 ± 4.6	nd
**6**	4-Chlorophenyl	74.6 ± 5.2	>100	81.4 ± 7.3	69.0 ± 8.5^c^
**7**	4-Bromophenyl	70.1 ± 1.5	nd	97.1 ± 5.5	nd
**8**	3-Fluorophenyl	72.8 ± 5.1	>100	73.3 ± 5.4	74.1 ± 1.2^c^
**9**	3-Chlorophenyl	52.5 ± 6.7	34.2 ± 6.4^a^	97.7 ± 4.8	>100
**10**	3-Bromophenyl	68.3 ± 4.8	nd	100.1 ± 4.4	nd
**11**	2-Fluorophenyl	50.3 ± 7.8	13.2 ± 3.5^a,b^	84.6 ± 3.2	34.7 ± 5.2^d^
**12**	2-Chlorophenyl	69.0 ± 6.4	nd	88.7 ± 3.6	nd
**13**	4-Methylphenyl	88.7 ± 4.2	nd	100.2 ± 5.3	nd
**14**	4-Methoxyphenyl	87.9 ± 7.5	nd	95.9 ± 6.4	nd
**15**	2-Furyl	104.5 ± 3.6	nd	100.1 ± 7.1	nd
**16**	2-Thiophenyl	99.4 ± 5.7	nd	95.6 ± 5.3	nd
**17**	3-Pyridyl	51.8 ± 2.5	15.7 ± 5.9^a,b^	75.7 ± 6.3	33.9 ± 7.1^d^
**18**	4-Pyridyl	61.3 ± 3.4	nd	108.7 ± 2.4	nd
**19**	1-Naphthyl	53.4 ± 4.3	14.3 ± 2.9^a,b^	88.9 ± 1.7	>100
**20**	1,4-Biphenyl	44.2 ± 3.3	22.7 ± 6.0^a,b^	95.9 ± 11.2	>100
**Dox**	–	nd	0.61 ± 0.2^b^	nd	1.03 ± 0.3^e^

a–e Different letters indicate different values with statistical significance *p*< 0.05 in Tukey’s multiple comparisons test; dox: doxorubicin (reference antineoplastic drug); nd: not determined; Cpd: compound.

In previous studies conducted by our group, chalcone with unsubstituted rings A and B had potent antiproliferative and pro-apoptotic activities^18^. However, its low solubility in water enables limited additional pharmacological assays, mainly *in vivo* experiments. Therefore, we designed a series of analogues with amino group at position 4’ on ring A, which had higher solubility in water compared to unsubstituted chalcone^16^. We evaluated chalcone with amino at position 4’ and unsubstituted ring B (**1**), which was inactive against MCF-7 (%MVC = 97.3 ± 6.0) and active against MDA-MB-231 (%MVC = 80.6 ± 4.0). Such result encouraged us to prepare further analogues with substitutions on ring B. Mai et al. reported EWG on ring B of chalcones improved antiproliferative activity against a broad panel of cancer cells^16^. We assayed analogues substituted by nitro (**2**), trifluoromethyl (**3**), cyano (**4**), and halogens (**5**–**12**). 3-Chloro-4’-aminochalcone (**9**) and 2-fluoro-4’-aminochalcone (**11**) were the two most active compounds against MCF-7 line, with %MVC rates of 52.5 ± 6.7 and 50.3 ± 7.8, respectively. 3-Fluoro-4’-aminochalcone (**8**) was the most potent against MDA-MB-231 line, with %MVC of 73.3 ± 5.4. In summary, comparison among %MVC values of chalcones with EWG on ring B and **1** suggested more electronegative halogens corroborated to antiproliferative activity. 4-Methyl-4’-aminochalcone (**13**) and 4-methoxy-4’-aminochalcone (**14**) were less active than halogenated-chalcones (**5**–**15**), corroborating negative effect of EDG towards bioactivity. We hypothesised rings containing six *π* electrons, similarly to phenyl ring of **1**, could conduce to bioactive analogues^31^. 2-Furyl-4’-aminochalcone (**15**) and 2-thiophenyl-4’-aminochalcone (**16**) were inactive compounds. Pyridylchalcones **17** and **18** were strongly active against MCF-7 line, with %MVC rates of 51.8 ± 2.5 and 61.3 ± 3.4, respectively. We also investigated the effect of additional benzene ring towards bioactivity, evaluating naphthylchalcone **19,** and biphenyl chalcone **20**, which have ten and twelve *π* electrons on ring B, respectively. Both chalcones presented strong antiproliferative activity against MCF-7 line, with %MVC rates of 53.4 ± 4.3 and 44.2 ± 3.3, respectively. Despite effect against both lines, MCF-7 line (ER) was more sensitive to chalcones than MDA-MB-231 line (TNBC) (Table 1).

The five most antiproliferative compounds against MCF-7 were **9**, **11**, **17**, **19**, and **20** (%MVC values lower than 60). Against MDA-MB-231, the five most potent ones were **1**, **6**, **8**, **11**, and **17** (%MVC values lower than 85). These seven compounds were selected to determine IC_50_ values for both cell lines (Table 1). Chalcones **1**, **6**, and **8** were inactive against MCF-7 (IC_50_ > 100 µM) and active against MDA-MB-231, with IC_50_ values of 60.3, 69.0, and 74.1 µM, respectively. Chalcones **19** and **20** were active against MCF-7 with IC_50_ values of 14.3 and 22.7 µM, respectively, and inactive against MDA-MB-231 (IC_50_ > 100 µM). Chalcones **11** and **17** were active against both cell lines, displaying IC_50_ values of 13.2–34.2 µM and were selected for additional bioassays.

### Antiproliferative activity of selected chalcones 11 and 17 under acidosis

In normal cells, intracellular and extracellular pH values are 7.2 and 7.4, respectively. On the other hand, in breast tumour cells, intracellular and extracellular pH values are 7.1–7.4 and 6.5–7.1, respectively^24^^,^^32^. This extracellular acidosis has been related to Warburg effect, in which tumour cells are in intense anaerobic metabolism, producing and exporting acid compounds through transporters. The biological central interest in acidosis is due to clear relationship with invasiveness and metastasis potential^24^^,^^33^. Tumour cells in acidic extracellular microenvironment can block membrane permeation of weak basic compounds, leading to ion-trapping effect. This phenomenon is caused by protonation of basic functionalities, converting neutral into cationic compounds. Cationic form exhibits reduced passive permeation through membrane phospholipids when compared to neutral form. Doxorubicin is antineoplastic and weak basic drug and has demonstrated low efficacy in acidosis microenvironment due to ion-trapping effect^34^.

**Figure 1.**
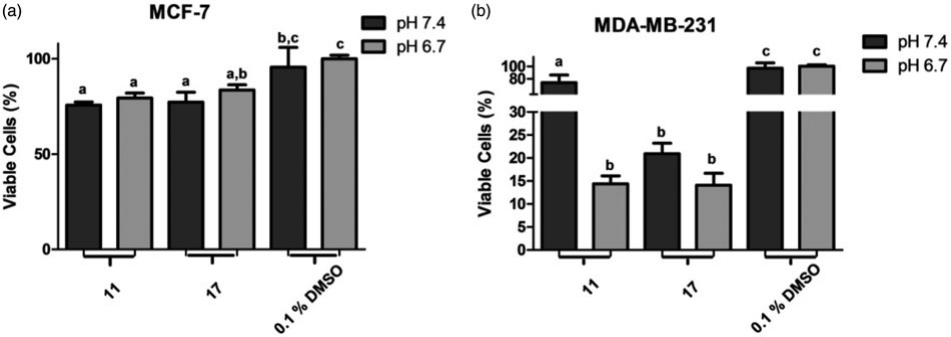
Percentage of metabolically viable cells (%MVC) under acidosis. (a) treatments with selected chalcones **11** and **17** against MCF-7 line; (b) treatments with selected chalcones **11** and **17** against MDA-MB-231 line. Different letters in the graph indicate statistical difference with significance *p*< 0.05 in Tukey’s multiple comparisons test. DMSO 0.1% was used as negative control.

Chalcones **11** and **17** were submitted to antiproliferative assays under acid microenvironment (at pH 6.7) against both breast cancer cell lines (Figure 1(a,b). In experiments with MCF-7 line, both chalcones maintained their antiproliferative effect, showing ion-trapping resistance. In MDA-MB-231 line, chalcone **17** maintained similar %MVC rate at both pH values. Interestingly, chalcone **11** was five times more active at pH 6.7 (acidosis) than at 7.4, with %MVC of 14.4 and 73.7, respectively.

### Pro-apoptotic activity of selected chalcones 11 and 17

**Figure 2.**
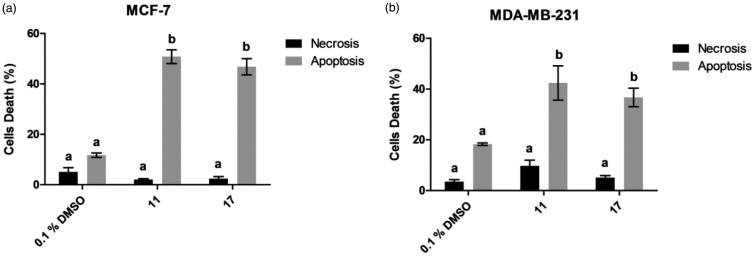
Pro-apoptotic activity of selected chalcones **11** and **17** towards MCF-7 (a) and MDA-MB-231 (b) cells. Different letters indicate statistical difference with significance *p*< 0.05 in Tukey’s multiple comparisons test. DMSO 0.1% was used as a vehicle control.

Antiproliferative compounds can induce tumour cell death by several mechanisms^35^. Among these, apoptosis and necrosis are the most common and studied ones. Apoptosis events have been induced by chalcones towards different cancer cell lines^36^^,^^37^. In order to investigate antiproliferative effect mechanism of selected chalcones **11** and **17** towards MCF-7 and MDA-MB-231 lines, Annexin V/PI staining and flow cytometry were used to detect whether cells were dying due to apoptosis or necrosis events (Figure 2).

Chalcones **11** and **17** induced 50.8% and 46.1% apoptotic events in MCF-7 line, 5- and 4-fold increases compared to cell treated with 0.1% DMSO, respectively. In MDA-MB-231 line, chalcones **11** and **17** caused death through apoptosis in 42.4 and 36.3% of cells, respectively. Additionally, selected chalcones induced apoptosis rather than necrosis.

Chalcones are known to induce apoptosis in several cancer cells, being able to up regulate more than 15 pro-apoptotic markers expression, such as Bad, Bax, Bid, Bim, CD40, Fas, IGFBP-5, IGFBP-6, p21, and sTNF-R116. Hsu and Bortolloto and their respective collaborators have demonstrated pro-apoptotic activity of unsubstituted chalcone against MCF-7 breast cancer cells^38^^,^^39^. These authors have described intrinsic apoptotic pathway induced by unsubstituted chalcone, with inhibition of Bcl-2 and induction of Bax apoptotic markers.

### Effect of selected chalcones 11 and 17 on Sp1 and p53 proteins expression

**Figure 3.**
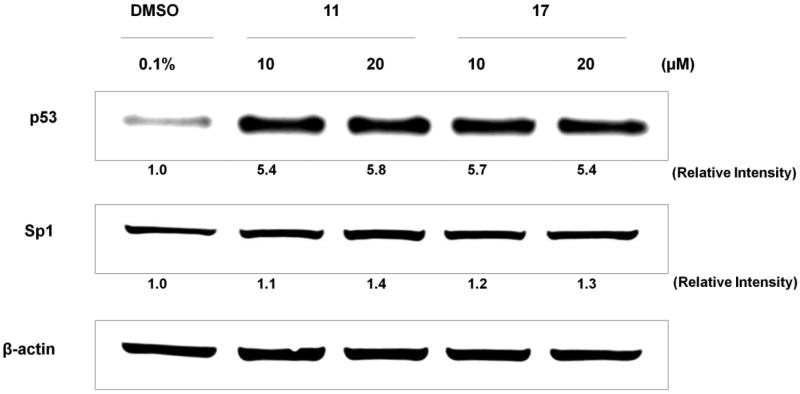
Effect of selected chalcones **11** and **17** on Sp1 and p53 proteins expression in MCF-7 cell line.

MCF-7 and MDA-MB-231 lines have demonstrated wild and mutant p53 protein, respectively^40^. Thus, we selected MCF-7 to conduct molecular target experiments. Western blot assay was performed to evaluate effect of chalcones **11** and **17** on p53 and Sp1 proteins expression in MCF-7 line for 24 h at 10 and 20 µM (Figure 3).

Sp1 protein is transcription factor involved in cell proliferation and differentiation. In breast cancers, it acts on invasion and metastasis processes^41^^,^^42^, and has been classified as marker for poor prognosis^43^. Chalcones **11** and **17** were not able to modulate Sp1 protein expression, at either concentration, presenting similar effect to DMSO 0.1% (negative control).

p53 protein is tumour suppressor and its mutated status has been related to several types of cancers. Expression of p53 in breast cancer cells varies according to its classification^44^^,^^45^. ER-positive and ER-negative cancer types have wild-type p53 and mutated protein forms, respectively^46^. Activation and stabilisation of wild-type p53 induce cell-cycle arrest and cell death through apoptosis, reducing cancer progression. This cell pathway has been recognised as attractive target to simple and low-weight-molecular compounds with promising antineoplastic potential. Chalcones **11** and **17** induced 5-fold upregulation of p53 expression in MCF-7 cells (Figure 3), indicating these compounds are able to activate and stabilise p53 protein expression. This result is the first evaluation of low-molecular-weight compounds against breast cancer cells. In this context, methoxychalcones and naphthylchalcones have been described as agents of p53 activation and stabilisation in prostate cancer and osteosarcoma cell lines, respectively^26^^,^^47^.

## Conclusions

In summary, we reported activity of series of 20 chalcones against two types of breast cancer cells, ER-positive (MCF-7 line) and TNBC (MDA-MB-231 line). Preliminary investigations suggested halogens on ring B and additional benzene rings play central role in antiproliferative activity. Basic chalcones **11** and **17** were antiproliferative agents under acidosis (at pH 6.7), displaying resistance to ion-trapping effect. These compounds induced apoptosis rather than necrosis in both cells, upregulating p53 expression in ER-positive cells (MCF-7 line).

## Supplementary Material

Supplemental Material
