# Supplementary material for: Antiproliferative activity and p53 upregulation effects of chalcones on human breast cancer cells
Source: J Enzyme Inhib Med Chem. 2019 May 23;34(1):1093–9. doi: 10.1080/14756366.2019.1615485 (PMC6534249; doi:10.1080/14756366.2019.1615485)
Supplement: Supplemental Material [file IENZ_A_1615485_SM0183.pdf]

Supplemental Data

Antiproliferative activity and p53 upregulation effects of chalcones on human breast cancer cells

Mariana Bastos dos Santos[1], Daiane Bertholin Anselmo[1], Jéssica Gisleine de Oliveira[2], Bruna V. Jardim-Perassi[2], Diego Alves Monteiro[3], Gabriel Silva[4], Eleni Gomes[3], Ana Lúcia Fachin[4], Mozart Marins[4], Débora Aparecida Pires de Campos Zuccari[2], Luis Octavio Regasini[1,*].

[1]Department of Chemistry and Environmental Sciences, Institute of Biosciences, Humanities and Exact Sciences (IBILCE), São Paulo State University (UNESP), 15054-000, São José do Rio Preto, São Paulo, Brazil.

[2]Department of Molecular Biology, Medicine College of São José do Rio Preto (FAMERP), 15090-000, São José do Rio Preto, São Paulo, Brazil.

[3]Department of Biology, Institute of Biosciences, Humanities and Exact Sciences (IBILCE), São Paulo State University (UNESP), 15054-000, São José do Rio Preto, São Paulo, Brazil.

[4]Biotechnology Unit, University of Ribeirão Preto (UNAERP), 14096-900, Ribeirão Preto, São Paulo, Brazil.

***Correspondence**: *luis.regasini@unesp.br (L.O.R.), Tel. +55-17-3221-2362.

1. SPECTROSCOPY DATA ANALYSES

1.1. (*E*)-4'-aminochalcone (1)

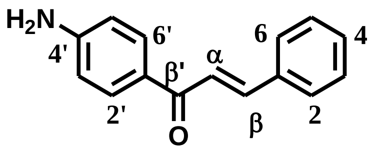

Yellow solid.

Purity: 99.3 %

UV-Vis: λ_{max} 306 and 364 nm.

Melting point, ^{1}H and ^{13}C NMR were represented in the supplementary materials of our previous work [1], and ^{1}H and ^{13}C NMR values were compared to other descriptions [2,3].

1.2. (*E*)-4-nitro-4'-aminochalcone (2)

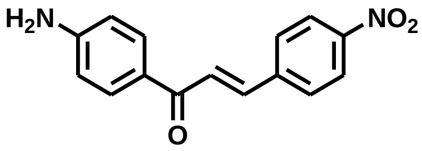

Orange solid.

Purity: 97.4 %

UV-Vis: λ_{max} 310 and 381 nm

Melting point, ^{1}H and ^{13}C NMR were represented in the supplementary materials of our previous work [1], and ^{1}H and ^{13}C NMR values were compared to other descriptions [2-4].

1.3. (*E*)-4-trifluoromethyl-4'-aminochalcone (3)

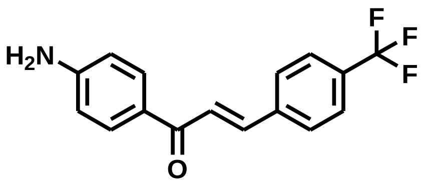

Yellow solid.

Yield: 96 %

Purity: 99.1 %

Melting Point: 129–130 °C

UV-Vis: λ_{max} 294 and 370 nm

^{1}H NMR (600 MHz, DMSO-d_6) δ_H: 8.07 (d, J = 8.3 Hz, H-3 and H-5), 8.01 (d, J = 15.6 Hz, H-β), 7.95 (d, J = 8.7 Hz, H-2' and H-6'), 7.79 (d, J = 8.3 Hz, H-2 and H-6), 7.66 (d, J = 15.6 Hz, H-α), 6.63 (d, J = 8.7 Hz, H-3' and H-5'), 6.23 (br s, 4'-N$\underline{H}_2$). **^{13}C NMR (150 MHz, DMSO-d_6) δ_C:** 186.0 (C-β'), 154.6 (C-4'), 139.9 (C-β), 139.7 (C-1), 131.8 (C-2' and C-6'), 130.0 ($J_{C,F}$ = 31.7 Hz, C-4) 129.5 (C-2 and C-6), 127.9 (C-1'), 126.1 (d, $J_{C,F}$ = 3.4 Hz, C-3 and C-5), 125.7 (C-α),124.6 ($J_{C,F}$ = 271.9 Hz, 4-CF_3) 113.2 (C-3' and C-5'). These ^{1}H and ^{13}C NMR values were compared to other descriptions [5].

1.4. (*E*)-4-cyano-4’-aminochalcone (4)

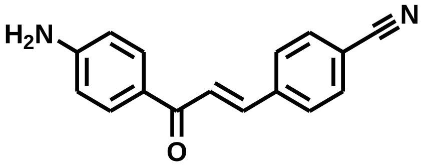

Yellow solid.

Yield: 95 %

Purity: 94.5 %

Melting Point: 202–204 °C

UV-Vis: λ_{max} 298 and 376 nm

^{1}H NMR (600 MHz, DMSO-d_6) δ_H: 8.05 (d, J = 8.7 Hz, H-2' and H-6'), 8.03 (d. J = 15,8 Hz, H-β), 7.95 (d, J = 8.6 Hz, H-3 and H-5), 7.91 (d, J = 8.6 Hz, H-2 and H-6), 7.64 (d, J = 15.8 Hz, H-α), 6.63 (d, J = 8.7 Hz, H-3' and H-5'), 6.24 (br s, 4'-N$\underline{H}_2$). **^{13}C NMR (150 MHz, DMSO-d_6) δ_C:** 186.0 (C-β'), 154.7 (C-4'), 140.3 (C-β), 139.7 (C-1),

133.1 (C-3 and C-5), 131.9 (C-2' and C-6'), 129.6 (C-2 and C-6), 126.3 (C-1'), 125.5 (C-α), 119.2 (4-CN), 113.2 (C-3' and C-5'), 112.2 (C-4). These ^{1}H and ^{13}C NMR values were compared to other descriptions [6,7].

1.5. (*E*)-4-fluoro-4'-aminochalcone (5)

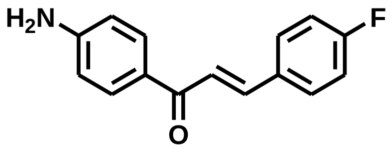

Yellow solid.

Purity: 94.1 %

UV-Vis: λ_{max} 310 and 363 nm

Melting point, ^{1}H and ^{13}C NMR were represented in the supplementary materials of our previous work [1], and ^{1}H and ^{13}C NMR values were compared to other descriptions [8].

1.6. (*E*)-4-chloro-4'-aminochalcone (6)

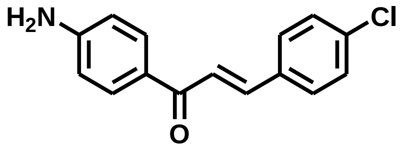

Yellow solid.

Purity: 97.9 %

UV-Vis: λ_{max} 310 and 367 nm

Melting point, ^{1}H and ^{13}C NMR were represented in the supplementary materials of our previous work [1], and ^{1}H and ^{13}C NMR values were compared to other descriptions [2,4,8,9].

1.7. (*E*)-4-bromo-4'-aminochalcone (7)

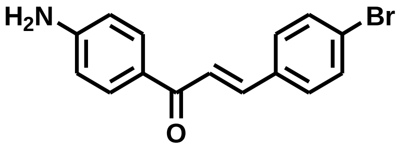

Yellow solid.

Yield: 75 %

Purity: 99.1 %

Melting Point: 149–150 °C

UV-Vis: λ_{max} 312 and 366 nm

^{1}H NMR (600 MHz, DMSO-*d*$_6$) δ_H: 7.93 (d, *J* = 8.8 Hz, H-2' and H-6'), 7.91 (d, *J* = 15.6 Hz, H-β), 7.81 (d, *J* = 8.5 Hz, H-2 and H-6), 7.64 (d, *J* = 8.5 Hz, H-3 and H-5), 7.58 (d, *J* = 15.6 Hz, H-α), 6.62 (d, *J* = 8.8 Hz, H-3' and H-5'), 6.18 (br s, 4'-N$\underline{H}_2$). **^{13}C NMR (150 MHz, DMSO-*d*$_6$) δ_C:** 186.1 (C-β'), 154.5 (C-4'), 140.5 (C-β), 135.0 (C-1), 132.2 (C-3 and C-5), 131.7 (C-2' and C-6'), 130.9 (C-2 and C-6), 125.7 (C-1'), 123.7 (C-α), 113.2 (C-3' and C-5'). These ^{1}H and ^{13}C NMR values were compared to other descriptions [10].

1.8. (*E*)-3-fluoro-4'-aminochalcone (8)[8,11]

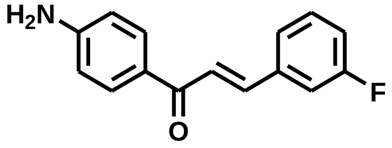

Yellow solid.

Yield: 30 %

Purity: 99.3 %

Melting Point: 126–127 °C

UV-Vis: λ_{max} 297 and 367 nm

^{1}H NMR (600 MHz, DMSO-*d*$_6$) δ_H: 7.96 (d, *J* = 8.7 Hz, H-2' and H-6'), 7.94 (d, *J* = 15.6 Hz, H-β), 7.81 (dd, *J* = 8.0 and 2.1 Hz, H-5), 7.65 (d, *J* = 8.0 Hz, H-6), 7.61 (d, *J* = 15.6 Hz, H-α), 7.48 (ddd, *J* = 8.0, 6.2 and 1.8 Hz, H-4), 7.25 (ddd, *J* = 8.4, 2.6 and 1.8 Hz, H-2), 6.63 (d, *J* = 8.8 Hz, H-3' and H-5'), 6.20 (br s, 4'-N$\underline{H}_2$). **^{13}C NMR (150 MHz, DMSO-*d*$_6$) δ_C:** 186.1 (C-β'), 163.0 (C-3, d, $J_{C,F}$ = 243.6), 154.5 (C-4'), 140.4 (C-β), 138.2 (C-1, d, $J_{C,F}$ = 8.0 Hz), 131.7 (C-2' and C-6'), 131.2 (C-5, d, *J* = 8.5 Hz), 125.7 (C-1'), 125.6 (C-6), 124.4 (C-α), 117.1 (C-4, d, $J_{C,F}$ = 21.5 Hz), 114.8 (C-2, d, $J_{C,F}$ = 21.9 Hz), 113.2 (C-3' and C-5'). These ^{1}H and ^{13}C NMR values were compared to other descriptions [8,11].

1.9. (*E*)-3-chloro-4'-aminochalcone (9)

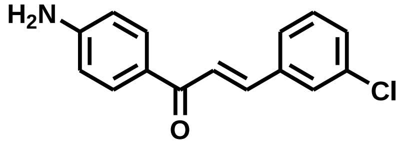

Yellow solid.

Yield: 44 %

Purity: 92.2 %

Melting Point: 140–141 °C

UV-Vis: λ_{max} 297 and 368 nm

^{1}H NMR (600 MHz, DMSO-*d*$_6$) δ_H: 8.03 (s, H-2), 7.96 (d, *J* = 8.7 Hz, H-2' and H-6'), 7.96 (d, *J* = 15.6 Hz, H-β), 7.80 – 7.73 (m, H-5), 7.59 (d, *J* = 15.6 Hz, H-α), 7.47 – 7.46 (m, H-4 and H-6), 6.63 (d, *J* = 8.7 Hz, H-3' and H-5'), 6.20 (br s, 4'-N$\underline{H}_2$). **^{13}C NMR (150 MHz, DMSO-*d*$_6$) δ_C:** 186.1 (C-β'), 154.5 (C-4'), 140.2 (C-β), 137.9 (C-1), 134.2 (C-3), 131.8 (C-2' and C-6'), 131.1 (C-5), 130.0 (C-4), 128.1 (C-1'), 128.0 (C-2), 125.6 (C-6), 124.5 (C-α), 113.2 (C-3' and C-5'). These ^{1}H and ^{13}C NMR values were compared to other descriptions [4,8].

1.10. (*E*)-3-bromo-4'-aminochalcone (10)

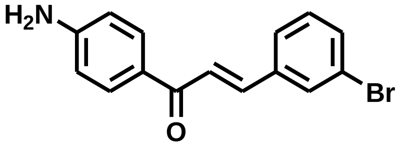

Yellow solid.

Yield: 76 %

Purity: 95.1 %

Melting Point: 165–166 °C

UV-Vis: λ_{max} 297 and 368 nm

^{1}H NMR (600 MHz, DMSO-d_6) δ_H: 8.17 (s, H-2), 7.96 (d, *J* = 8.5 Hz, H-2' and H-6'), 7.96 (d, *J* = 15.9 Hz, H-β), 7.81 (d, *J* = 7.8 Hz, H-6), 7.60 (d, *J* = 8.6 Hz, H-4), 7.57 (d, *J* = 15.9 Hz, H-α), 7.39 (dd, *J* = 8.6 and 7.8 Hz, H-5), 6.63 (d, *J* = 8.5 Hz, H-3' and H-5'), 6.20 (br s, 4'-$\mathrm{N\underline{H}_2}$). **^{13}C NMR (150 MHz, DMSO-d_6) δ_C:** 186.1 (C-β'), 154.5 (C-4'), 140.1 (C-β), 138.2 (C-1), 132.9 (C-4), 131.8 (C-2' and C-6'), 131.3 (C-5), 130.9 (C-2), 128.4 (C-1'), 125.6 (C-6), 124.5 (C-3), 122.8 (C-α), 113.2 (C-3' and C-5'). These ^{1}H and ^{13}C NMR values were compared to other descriptions [12].

1.11. (*E*)-2-fluoro-4'-aminochalcone (11)

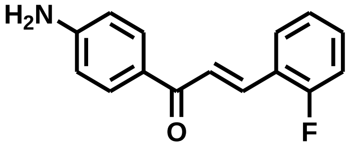

Yellow solid.

Yield: 39 %

Purity: 97.6 %

Melting Point: 116–117 °C

UV-Vis: λ_{max} 298 and 366 nm

^{1}H NMR (600 MHz, DMSO-d_6) δ_H: 8.08 (dd, J = 7.7 and 1.3 Hz, H-6), 7.92 (d, J = 8.7 Hz, H-2' and H-6'), 7.92 (d, J = 15.7 Hz, H-β), 7.73 (d, J = 15.7 Hz, H-α), 7.52 – 7.44 (m, H-4), 7.33 – 7.39 (m, H-3 and H-5), 6.63 (d, J = 8.7 Hz, H-3' and H-5'), 6.22 (br s, 4'-NH_2). **^{13}C NMR (150 MHz, DMSO-d_6) δ_C:** 186.0 (C-β'), 161.2 (C-2, d, J = 250.6 Hz), 154.6 (C-4'), 133.0 (C-β), 132.4 (C-4, d, $J_{C,F}$ = 8.6 Hz), 131.7 (C-2' and C-6'), 129.3 (C-1'), 125.4 (C-1, d, $J_{C,F}$ = 16.2 Hz), 125.3 (C-α) 125.0 (C-5), 123.2 (C-6, d, $J_{C,F}$ = 11.4 Hz), 116.5 (C-3, d, $J_{C,F}$ = 21.8 Hz), 113.2 (C-3' and C-5'). These ^{1}H and ^{13}C NMR values were compared to other descriptions [8,11].

1.12. (*E*)-2-chloro-4'-aminochalcone (12)

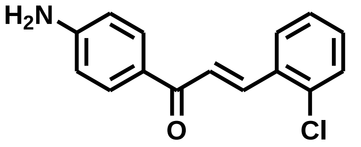

Yellow solid.

Yield: 43 %

Purity: 92.5 %

Melting Point: 135–136 °C

UV-Vis: λ_{max} 297 and 368 nm

^{1}H NMR (600 MHz, DMSO-d_6) δ: 8.18 (dd, J = 7.1 and 2.3 Hz, H-6), 7.95 (d, J = 8.6 Hz, H-2' and H-6'), 7.94 (d, J = 15.2 Hz, H-β), 7.94 (dd, J = 9.7 and 7,1 Hz. H-5), 7.57 – 7.54 (m, H-4), 7.44 (dd, J = 6.6 and 3.1 Hz, H-3), 7.43 (d, J = 15.2 Hz, H-α), 6.64 (d, J = 8.6 Hz, H-3' and H-5'), 6.23 (br s, 4'-$N\underline{H}_2$). **^{13}C NMR (150 MHz, DMSO-d_6) δ:** 185.9 (C-β'), 154.6 (C-4'), 136.5 (C-β), 134.5 (C-1), 133.3 (C-4), 131.8 (C-2' and C-6'), 131.8 (C-2), 130.4 (C-3), 128.8 (C-1'), 128.1 (C-6), 125.7 (C-α), 125.5 (C-5), 113.2 (C-3' and C-5'). These ^{1}H and ^{13}C NMR values were compared to other descriptions [4].

1.13. (*E*)-4-methyl-4'-aminochalcone (13)

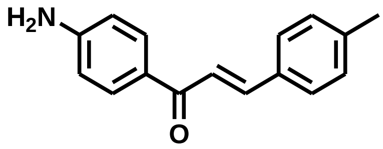

Light yellow solid.

Purity: 99.2 %

UV-Vis: λ_{max} 364 nm

Melting point, ^{1}H and ^{13}C NMR were represented in the supplementary materials of our previous work [1], and ^{1}H and ^{13}C NMR values were compared to other descriptions [2,8].

1.14. (*E*)-4-methoxy-4'-aminochalcone (14)

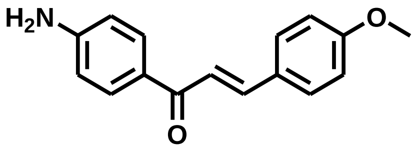

Light yellow solid.

Purity: 99.7 %

UV-Vis: λ_{max} 369 nm

Melting point, ^{1}H and ^{13}C NMR were represented in the supplementary materials of our previous work [1], and ^{1}H and ^{13}C NMR values were compared to other descriptions [3,8].

1.15. (*E*)-2-furyl-4'-aminochalcone (15)

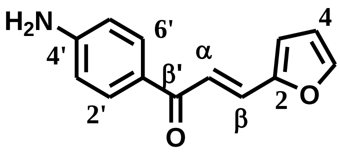

Orange solid.

Purity: 99.8 %

UV-Vis: λ_{max} 372 nm

Melting point, ^{1}H and ^{13}C NMR were represented in the supplementary materials of our previous work [1], and ^{1}H and ^{13}C NMR values were compared to other descriptions [8,13].

1.16. (*E*)-2-thiophenyl-4'-aminochalcone (16)

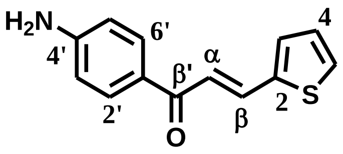

Yellow solid.

UV-Vis: λ_{max} 262 and 373 nm

Purity: 97.1 %

Melting point, ^{1}H and ^{13}C NMR were represented in the supplementary materials of our previous work [1], and ^{1}H and ^{13}C NMR values were compared to other descriptions [8].

1.17. (*E*)-2-pyridyl-4'-aminochalcone (17)

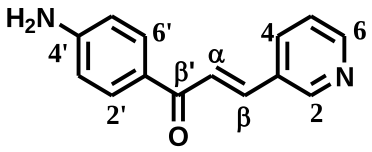

Light orange solid.

Yield: 63 %

Purity: 90.6 %

Melting Point: 179–180 °C

UV-Vis: λ_{max} 296 and 367 nm

^{1}H NMR (600 MHz, DMSO-d_6) δ_H: 8.98 (d, J = 1.3 Hz, H-6), 8.59 (dd, J = 1.2 and 4.0, H-2), 8.32 (d, J = 7.9 Hz, H-4), 8.02 (d, J = 15.7 Hz, H-β), 7.96 (d, J = 8.6 Hz, H-2'

and H-6'), 7.64 (d, *J* = 15.7 Hz, H-α), 7.47 (dd, *J* = 7.8 and 7.8 Hz, H-5), 6.63 (d, *J* = 8.6 Hz, H-3' and H-5'), 6.21 (br s, 4'-N$\underline{H}_2$). **^{13}C NMR (150 MHz, DMSO-d_6) δ_C:** 186.0 (C-β'), 154.6 (C-4'), 150.9 (C-2), 150.5 (C-6), 138.4 (C-β), 135.2 (C-4), 131.8 (C-2' and C-6'), 131.4 (C-4), 125.5 (C-1'), 124.8 (C-α), 124.3 (C-5). 113.2 (C-3' and C-5'). These ^{1}H and ^{13}C NMR values were compared to other descriptions [8,14].

1.18. (*E*)-4-pyridyl-4'-aminochalcone (18)

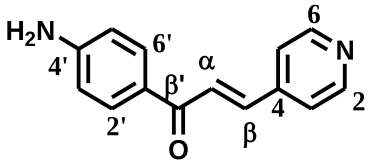

Yellow solid.

Yield: 65 %

Purity: 91.2 %

Melting Point: 166–167 °C

UV-Vis: λ_{max} 296 and 367 nm

^{1}H NMR (600 MHz, DMSO-d_6) δ_H: 8.64 (d, *J* = 6.0 Hz, H-6), 8.10 (d, *J* = 15.6 Hz, H-β), 7.95 (d, *J* = 8.7 Hz, H-2' and H-6'), 7.80 (d, *J* = 6.1 Hz, H-3), 7.56 (d, *J* = 15.6 Hz, H-α), 6.63 (d, *J* = 8.8 Hz, H-3' and H-5'), 6.27 (br s, 4'-N$\underline{H}_2$). **^{13}C NMR (150 MHz, DMSO-d_6) δ_C:** 185.9 (C-β'), 154.7 (C-4'), 150.7 (C-2 and C-6), 142.8 (C-β), 139.0 (C-4), 131.9 (C-2' and C-6'), 127.3 (C-α), 125.3 (C-1'), 122.8 (C-3 and C-5), 113.2 (C-3' and C-5'). These ^{1}H and ^{13}C NMR values were compared to other descriptions [8,14].

1.19. (*E*)-1-naphthyl-4'-aminochalcone (19)

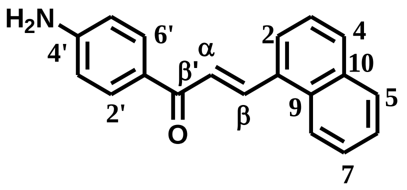

Yellow solid.

Purity: 99.9 %

UV-Vis: λ_{max} 378 nm

Melting point, ^{1}H and ^{13}C NMR were represented in the supplementary materials of our previous work [1], and ^{1}H and ^{13}C NMR values were compared to other descriptions [8,14].

1.20. (*E*)-4-pyridyl-4’-aminochalcone (20)

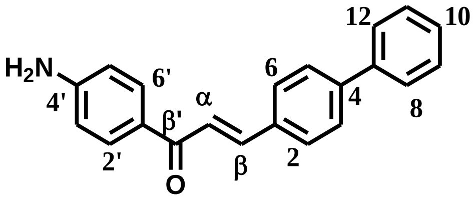

Light yellow solid.

Yield: 41 %

Purity: 99.0 %

Melting Point: 189–190 °C

UV-Vis: λ_{max} 369 nm

^{1}H NMR (600 MHz, DMSO-d_6) δ_H: 7.96 (d, J = 8.7 Hz, H-2' and H-6'), 7.94 (d, J = 8.0 Hz, H-3 and H-5), 7.93 (d, J = 15.5 Hz, H-β), 7.76 (d, J = 8.0 Hz, H-2 and H-6), 7.75 (dd, J = 8.0 and 1.1 Hz, H-8 and H-12), 7.67 (d, J = 15.5 Hz, H-α), 7.50 (dd, J = 7.7 and 7.4 Hz, H-9 and H-11), 7.41 (ddd, J = 7.4, 7.4 and 1.1 Hz, H-10), 6.63 (d, J = 8.7 Hz, H-3' and H-5'), 6.19 (br s, 4'-N$\underline{H}_2$). **^{13}C NMR (150 MHz, DMSO-d_6) δ_C:** 186.3 (C-β'), 154.4 (C-4'), 141.9 (C-β), 141.4 (C-7), 139.8 (C-4), 134.8 (C-1), 131.6 (C-2' and C-6'), 129.7 (C-9 and C-11), 129.5 (C-8 and C-12), 128.4 (C-1'), 127.5 (C-3 and C-5), 127.2

(C-2 and C-6), 125.8 (C-10), 122.9 (C-α), 113.2 (C-3' and C-5'). These ^{1}H and ^{13}C NMR values were compared to other descriptions [3].

Figure S.1. UV-vis spectrum of chalcone **1** from HPLC-DAD experiment

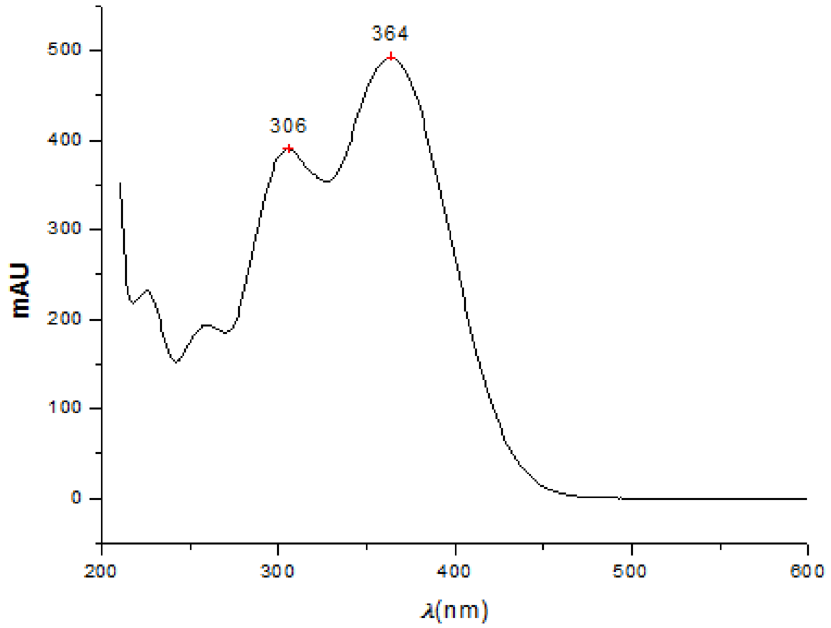

Figure S.2. HPLC-DAD chromatogram of chalcone **1**, MeOH:H_2O (3:1)

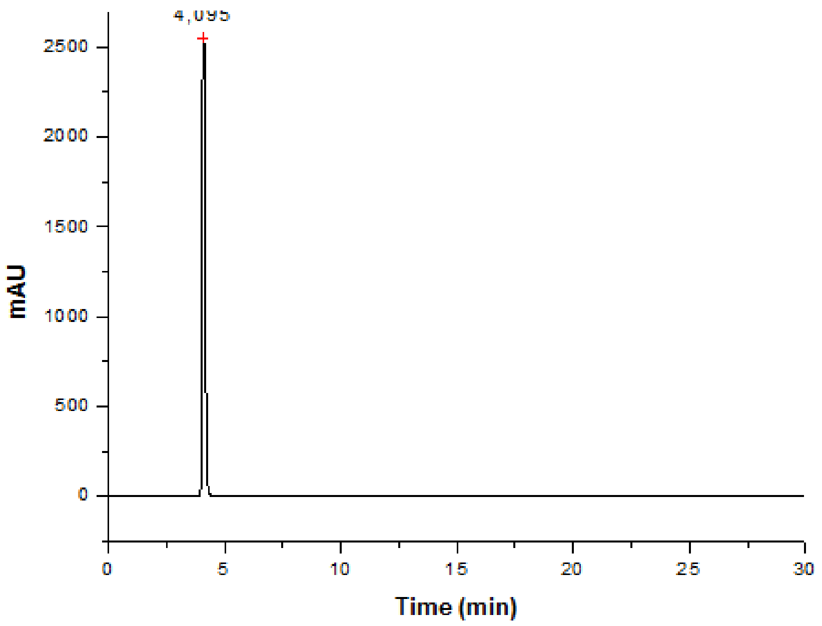

Figure S.3. UV-vis spectrum of chalcone **2** from HPLC-DAD experiment

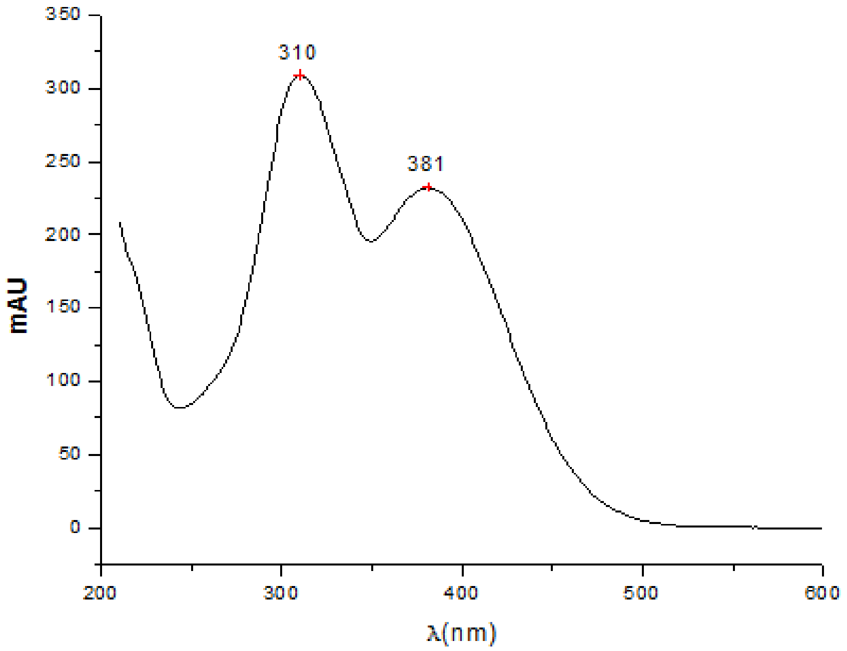

Figure S.4. HPLC-DAD chromatogram of chalcone **2**, MeOH:H_2O (3:1)

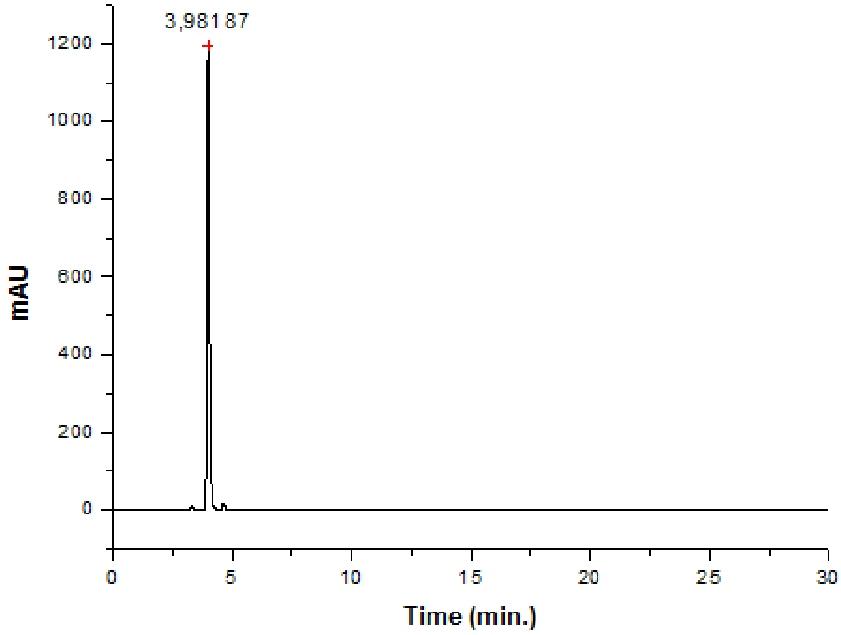

Figure S.5. ^{1}H NMR spectrum (DMSO-d_6, 600 MHz) of chalcone **3**

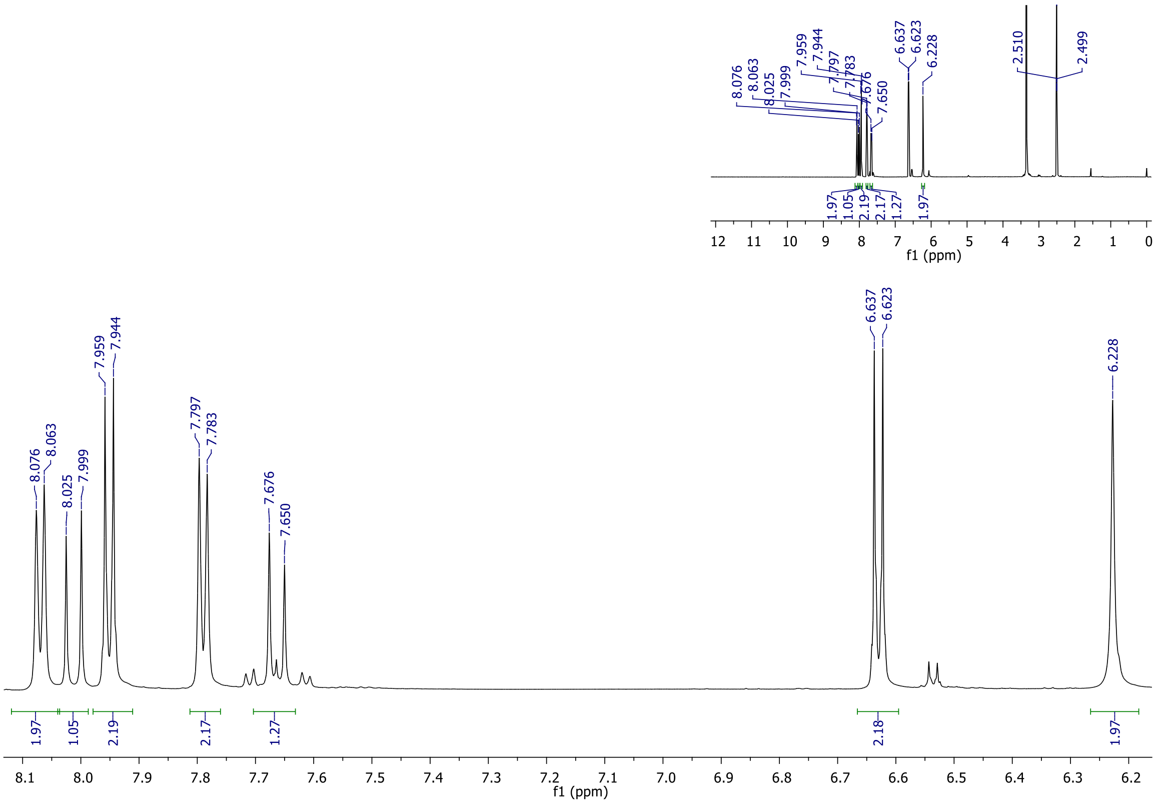

Figure S.6. ^{13}C NMR spectrum (DMSO-d_6, 150 MHz) of chalcone **3**

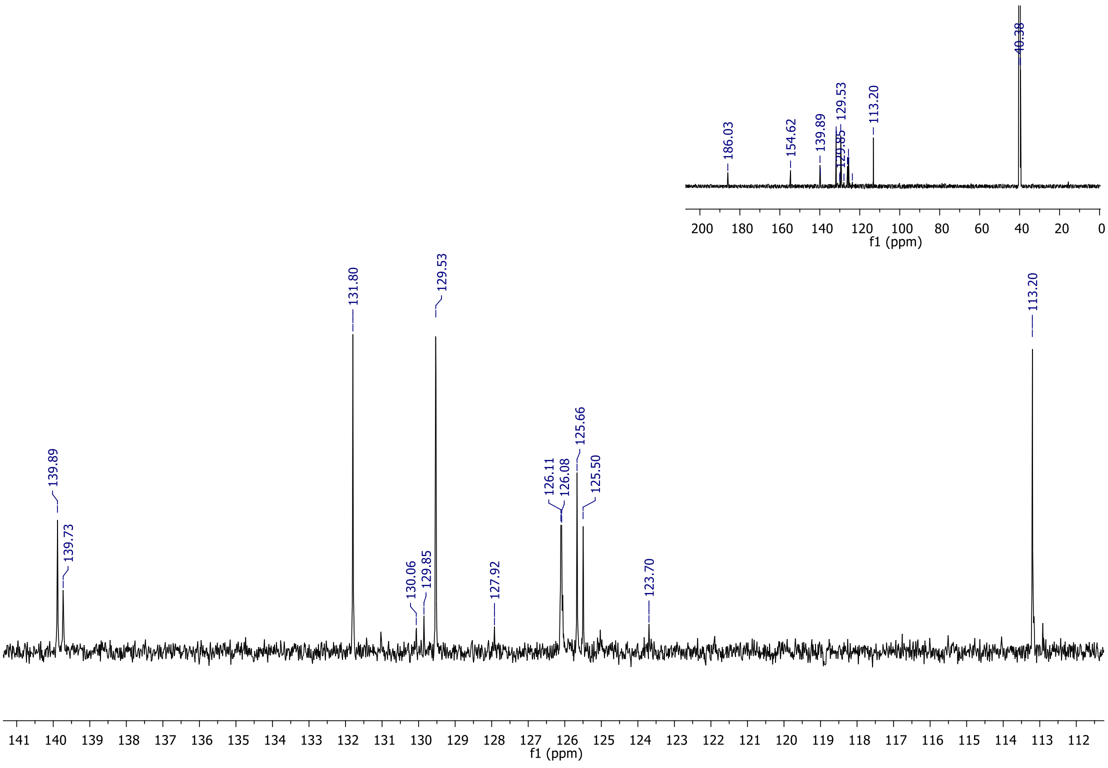

Figure S.7. UV-vis spectrum of chalcone **3** from HPLC-DAD experiment

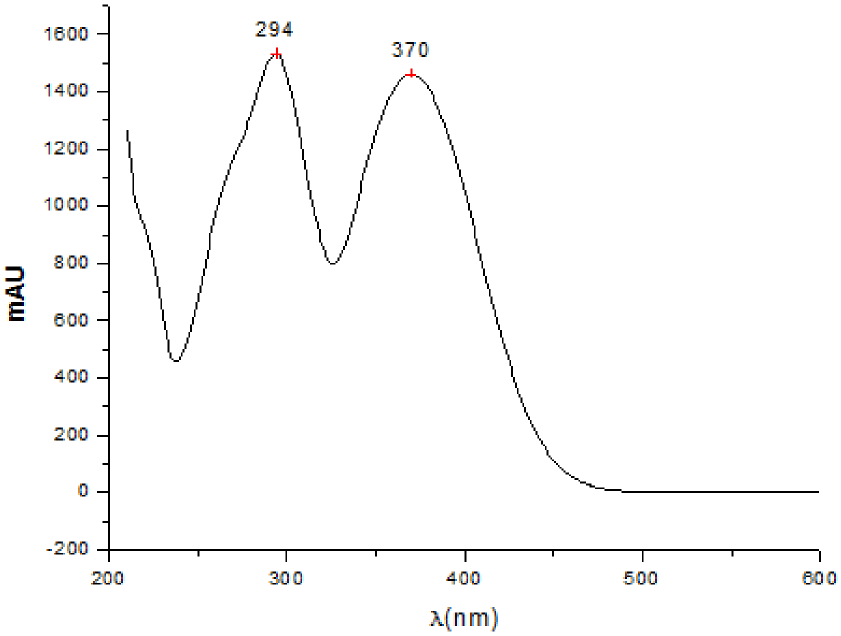

Figure S.8. HPLC-DAD chromatogram of chalcone **3**, MeOH:H_2O (3:1)

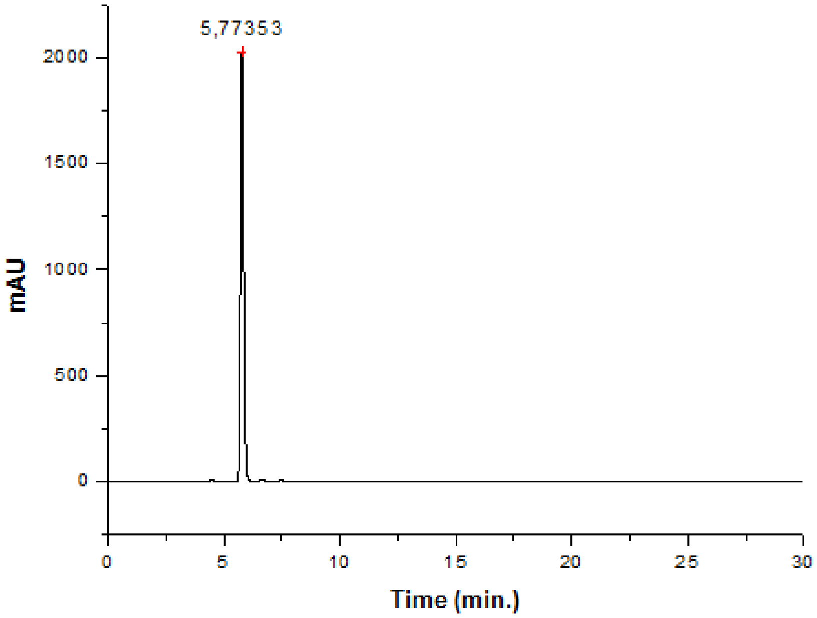

Figure S.9. ^{1}H NMR spectrum (DMSO-d_6, 600 MHz) of chalcone **4**

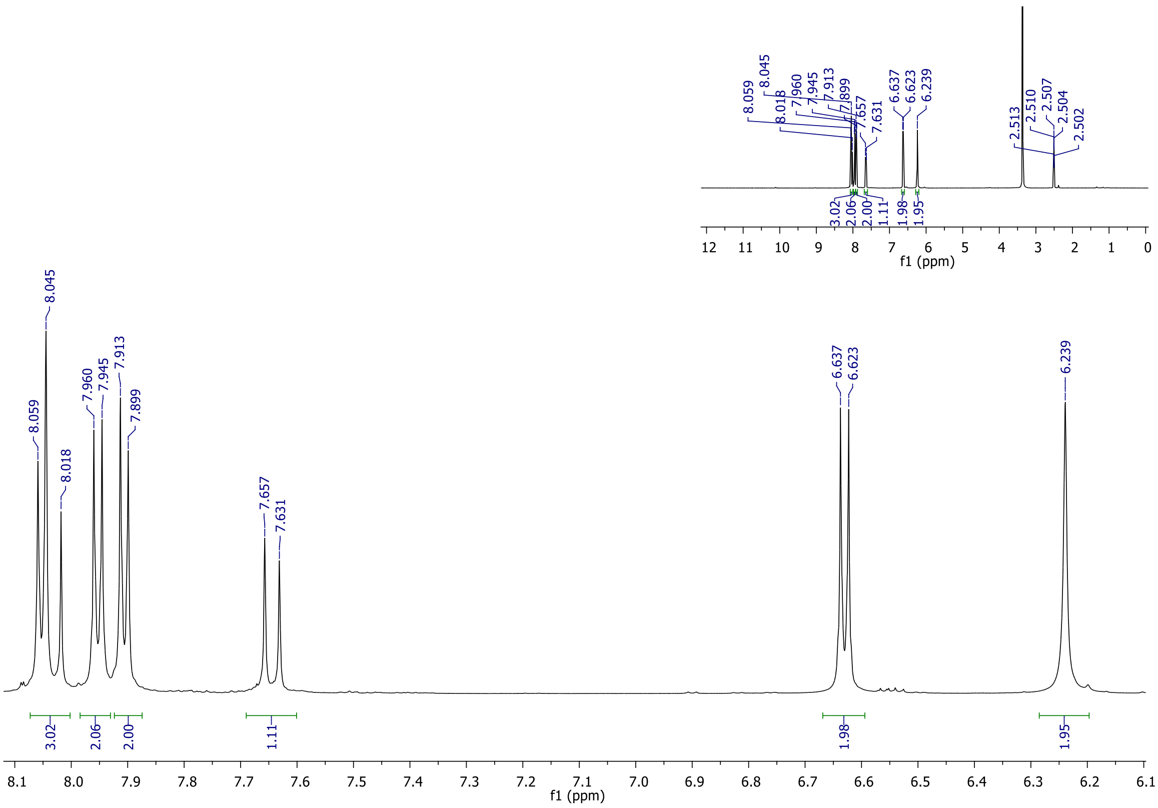

Figure S.10. ^{13}C NMR spectrum (DMSO-d_6, 150 MHz) of chalcone **4**

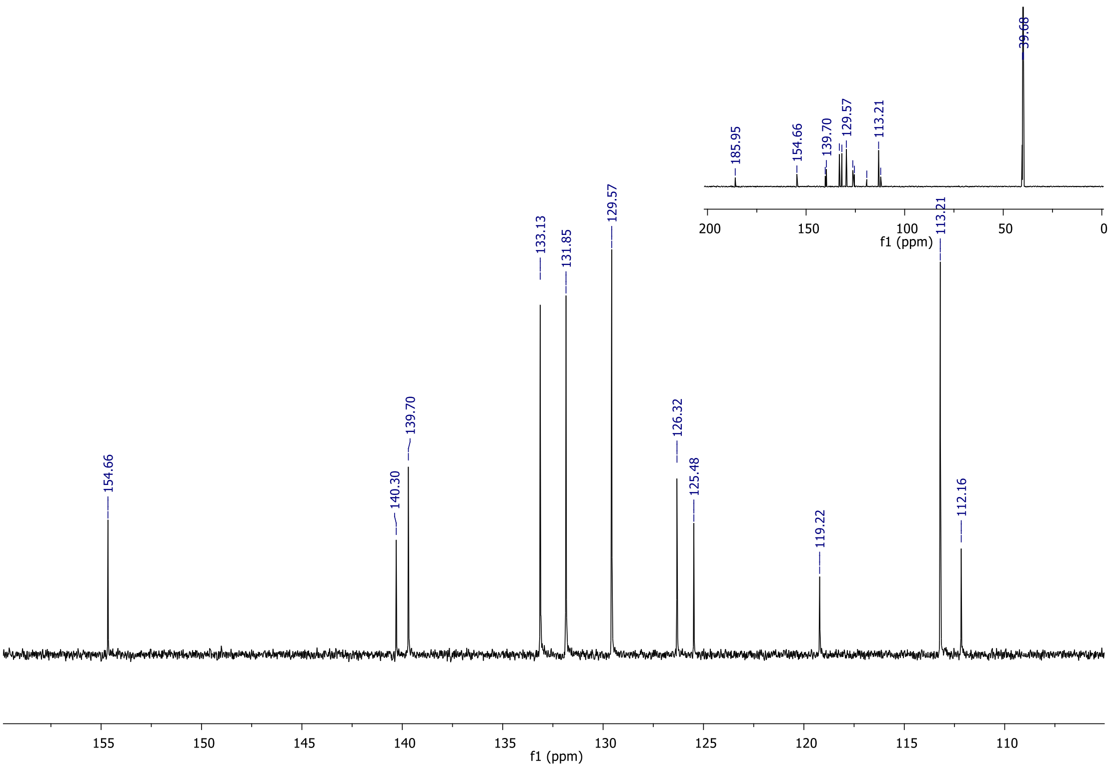

Figure S.11. UV-vis spectrum of chalcone **4** from HPLC-DAD experiment

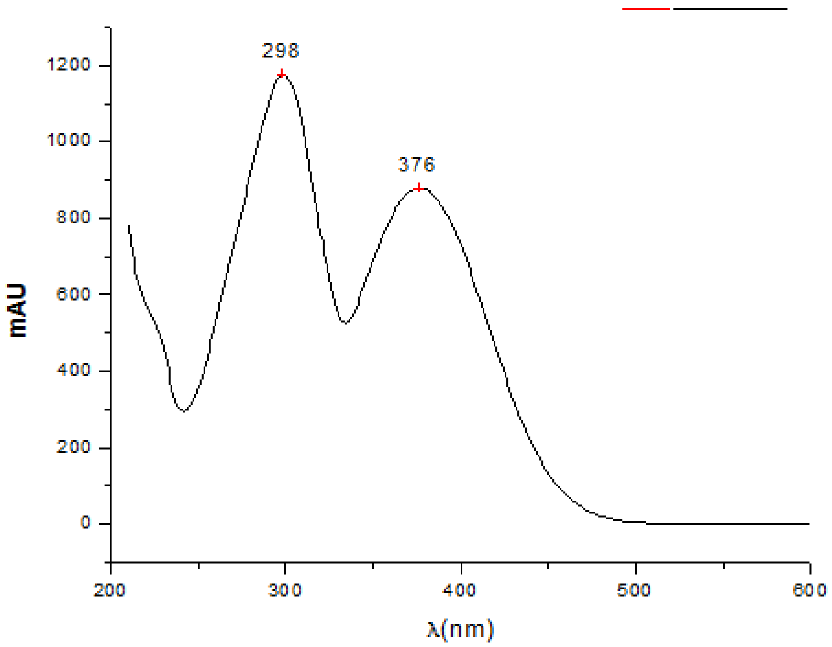

Figure S.12. HPLC-DAD spectrum of chalcone **4**, MeOH:H_2O (3:1)

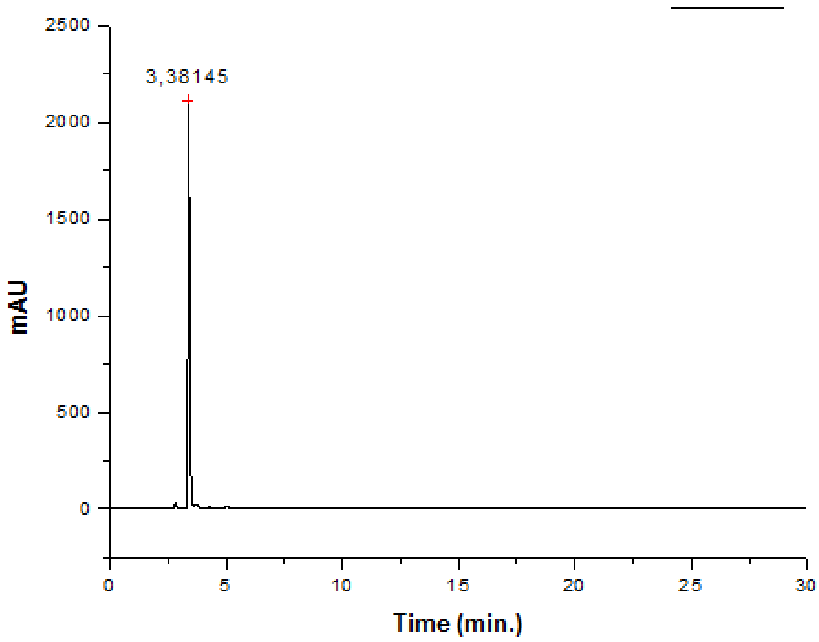

Figure S.13. UV-vis spectrum of chalcone **5** from HPLC-DAD experiment

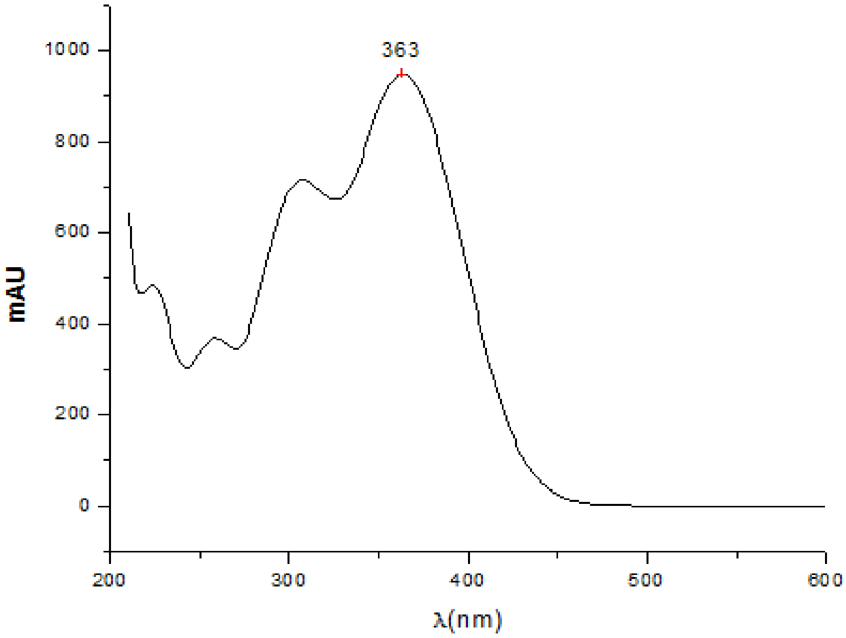

Figure S.14. HPLC-DAD chromatogram of chalcone **5**, MeOH:H_2O (3:1)

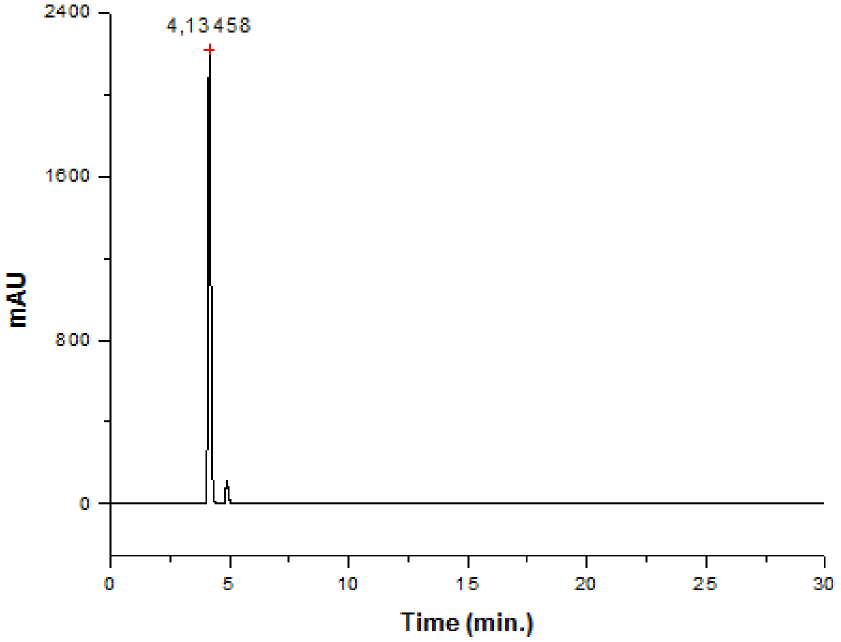

Figure S.15. UV-vis spectrum of chalcone **6** from HPLC-DAD experiment

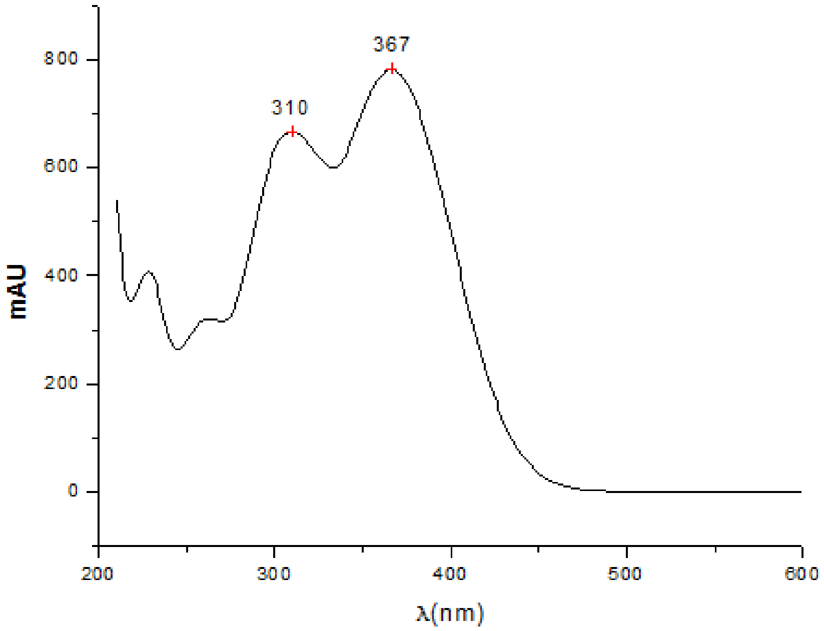

Figure S.16. HPLC-DAD chromatogram of chalcone **6**, MeOH:H_2O (3:1)

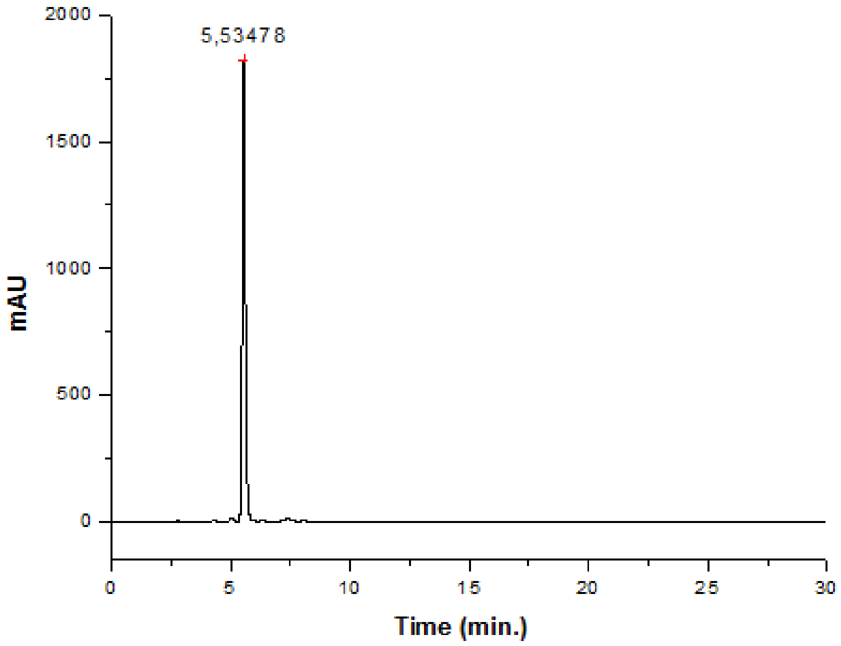

Figure S.17. ^{1}H NMR spectrum (DMSO-d_6, 600 MHz) of chalcone **7**

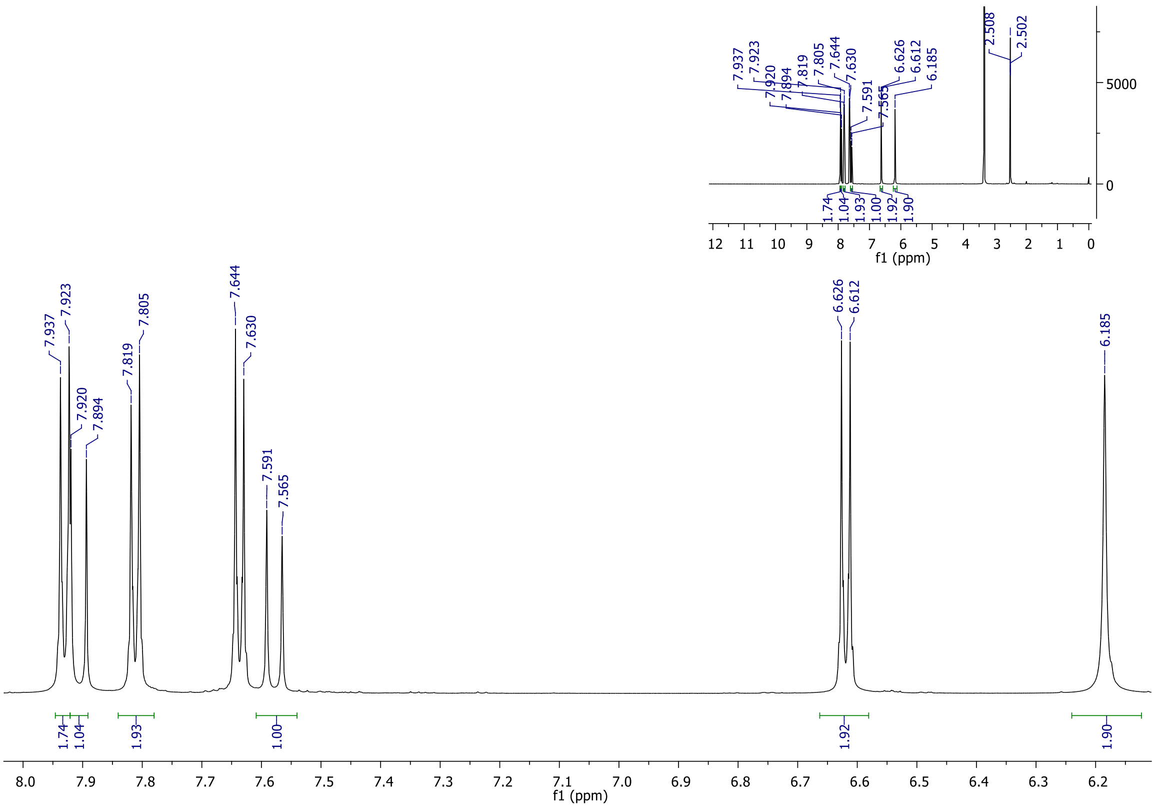

Figure S.18. ^{13}C NMR spectrum (DMSO-d_6, 150 MHz) of chalcone **7**

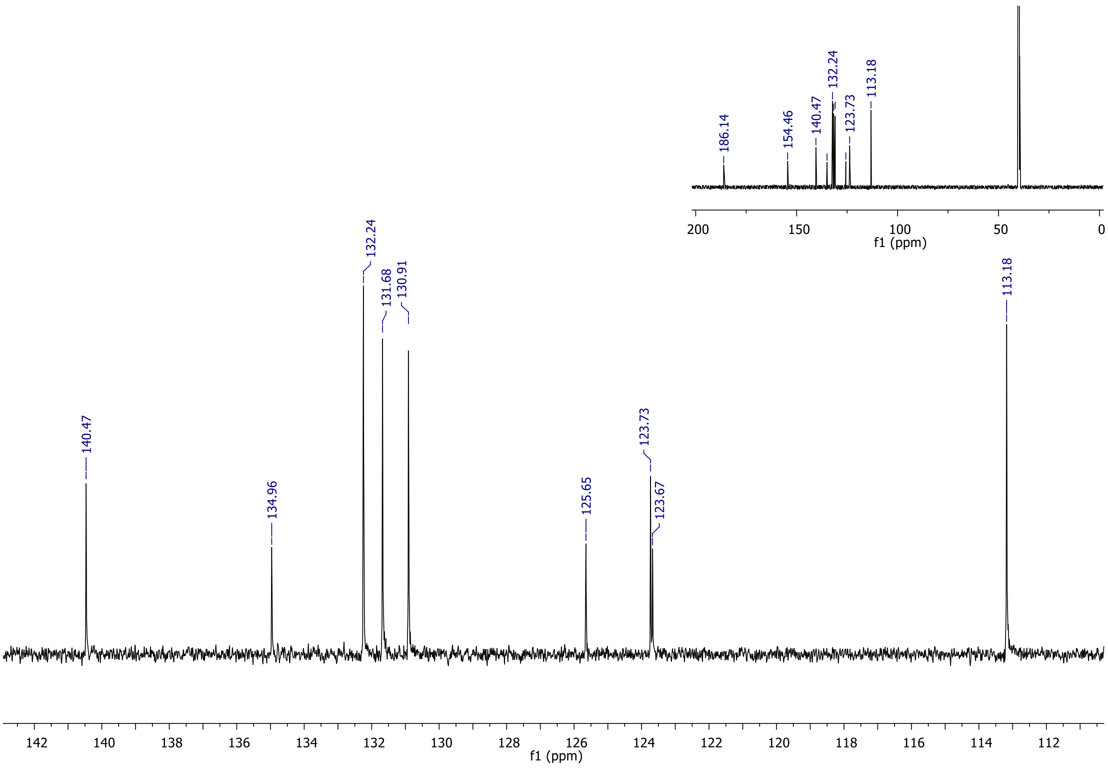

Figure S.19. UV-vis spectrum of chalcone **7** from HPLC-DAD experiment

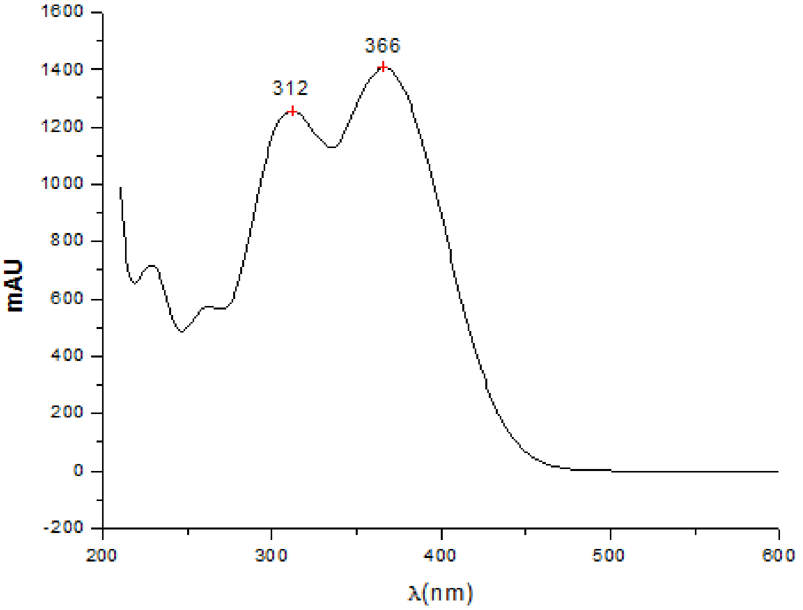

Figure S.20. HPLC-DAD chromatogram of chalcone **7**, MeOH:H_2O (3:1)

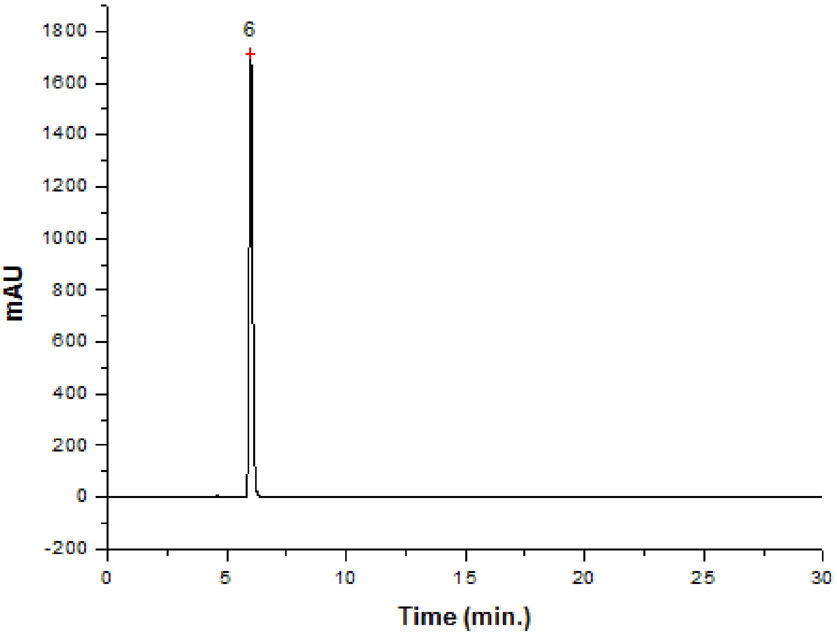

Figure S.21. ^{1}H NMR spectrum (DMSO-d_6, 600 MHz) of chalcone **8**

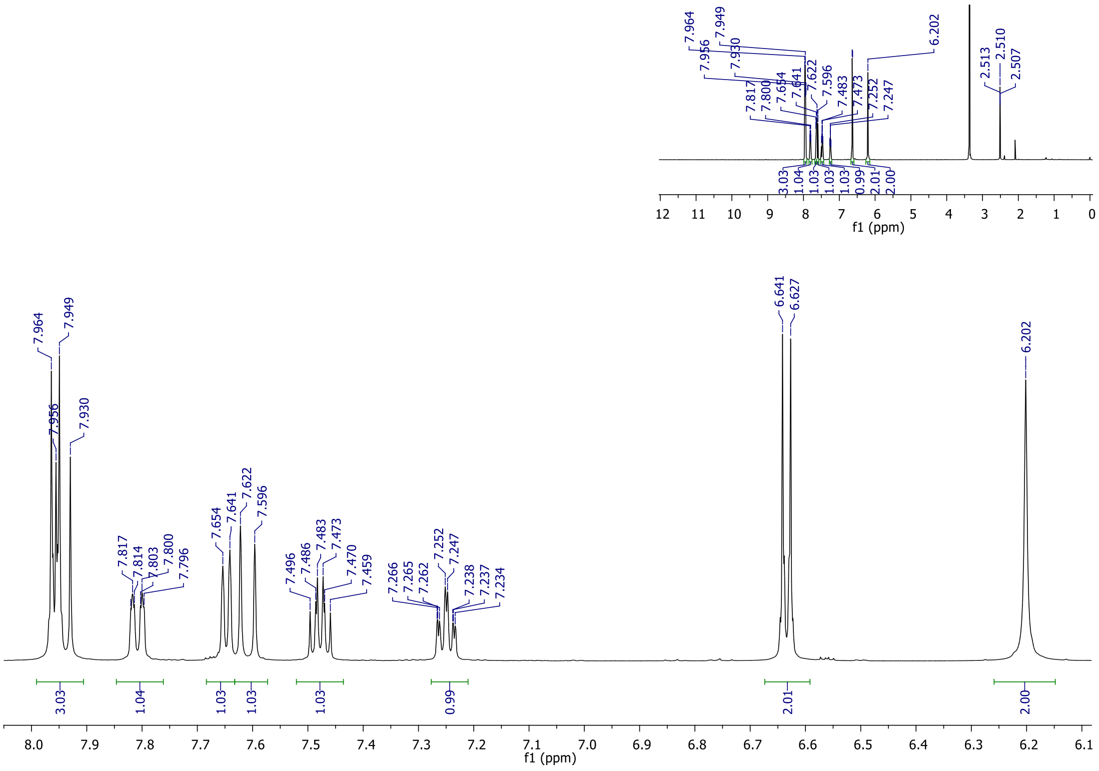

Figure S.22. ^{13}C NMR spectrum (DMSO-d_6, 150 MHz) of chalcone **8**

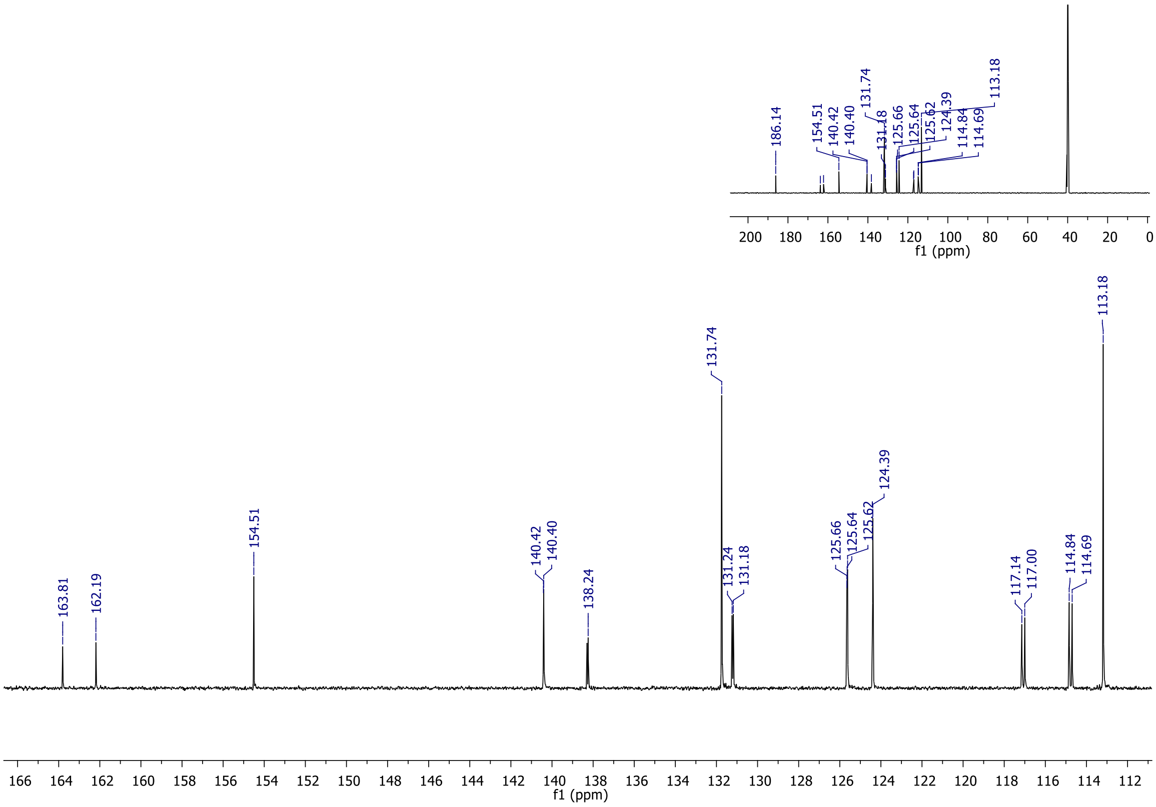

Figure S.23. UV-vis spectrum of chalcone **8** from HPLC-DAD experiment

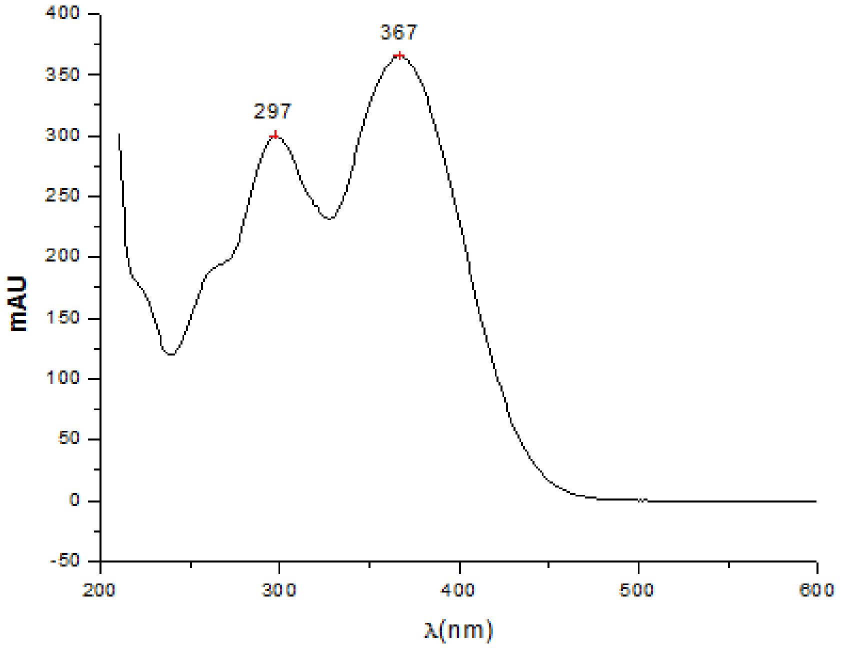

Figure S.24. HPLC-DAD chromatogram of chalcone **8**, MeOH:H_2O (3:1)

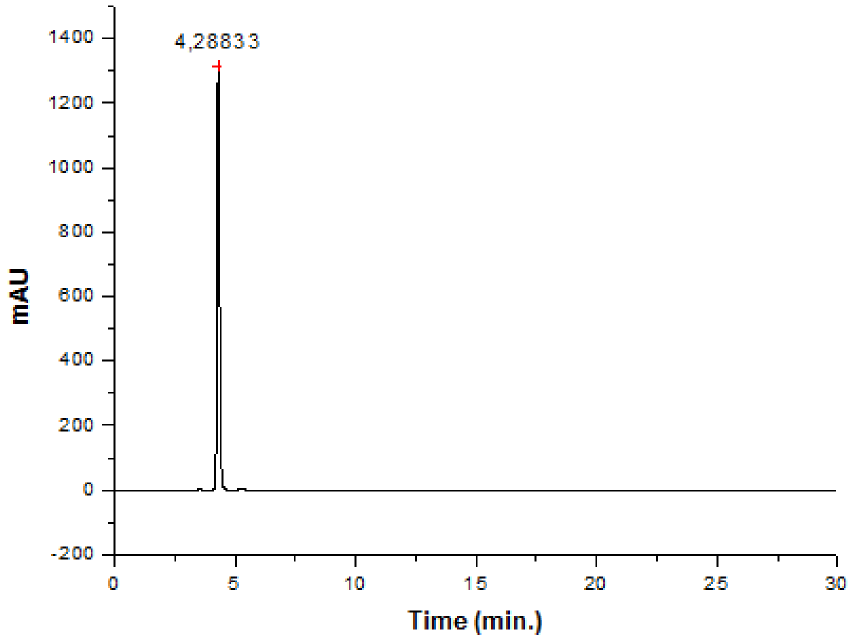

Figure S.25. ^{1}H NMR spectrum (DMSO-d_6, 600 MHz) of chalcone **9**

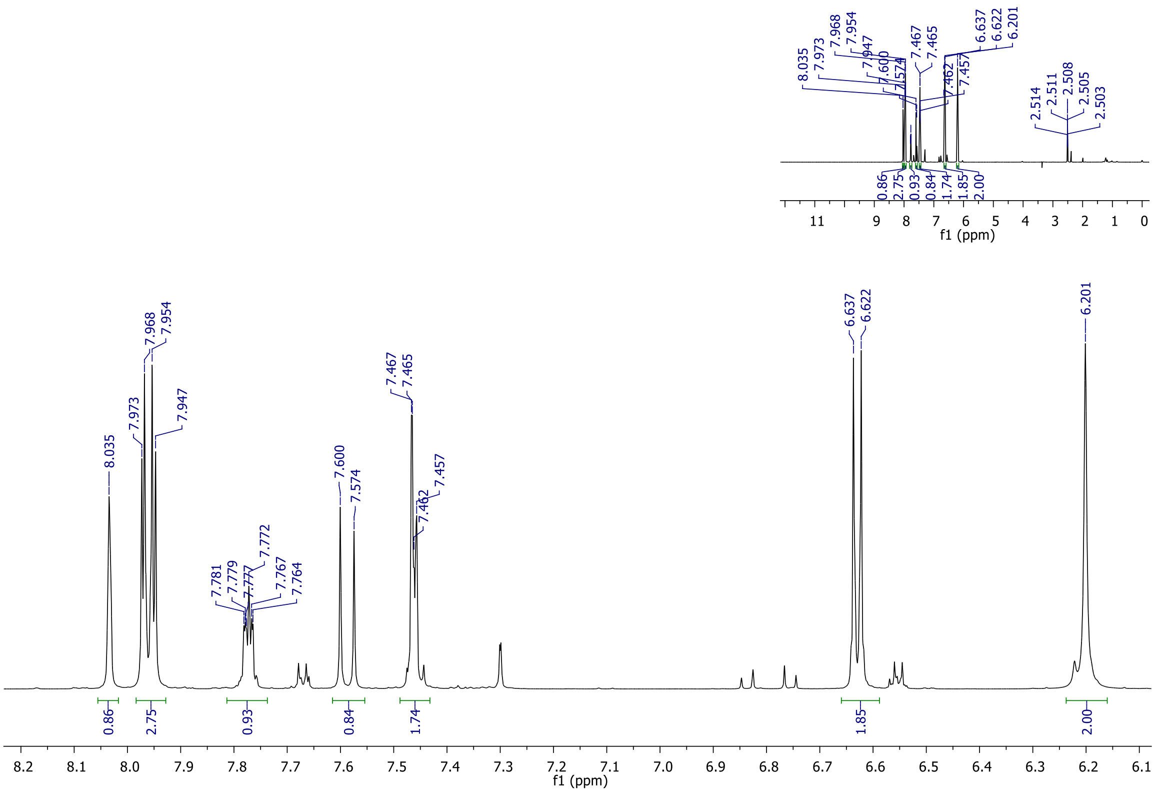

Figure S.26. ^{13}C NMR spectrum (DMSO-d_6, 150 MHz) of chalcone **9**

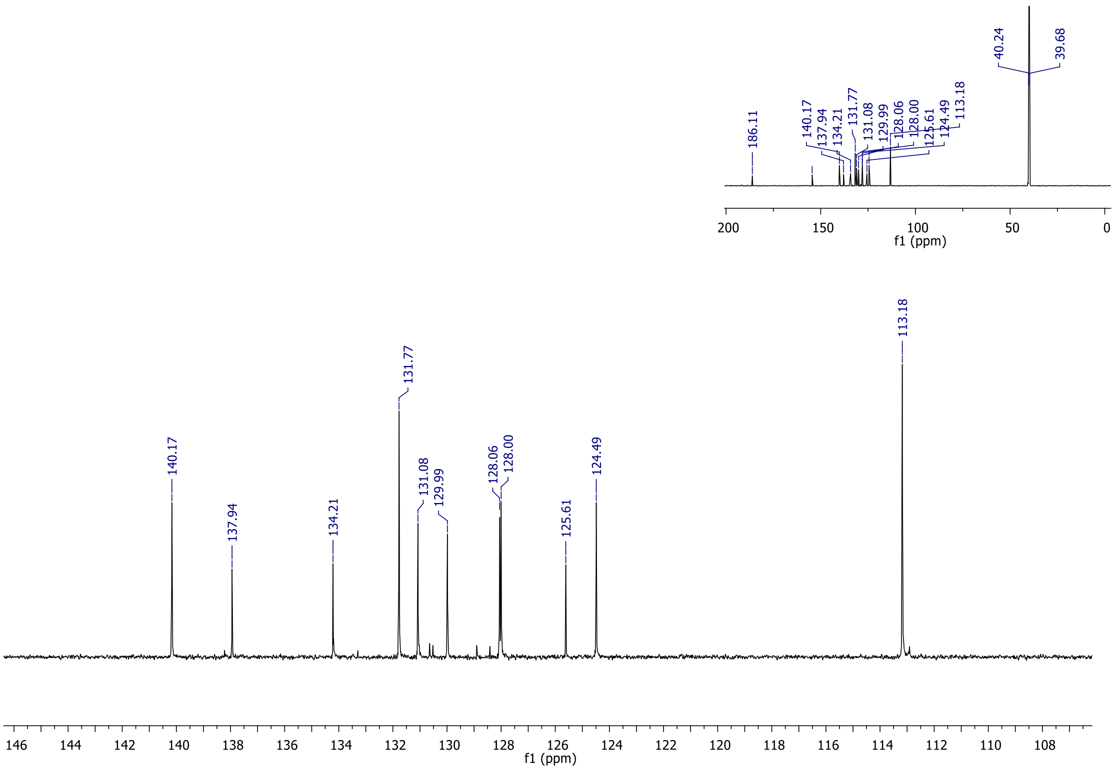

Figure S.27. UV-vis spectrum of chalcone **9** from HPLC-DAD experiment

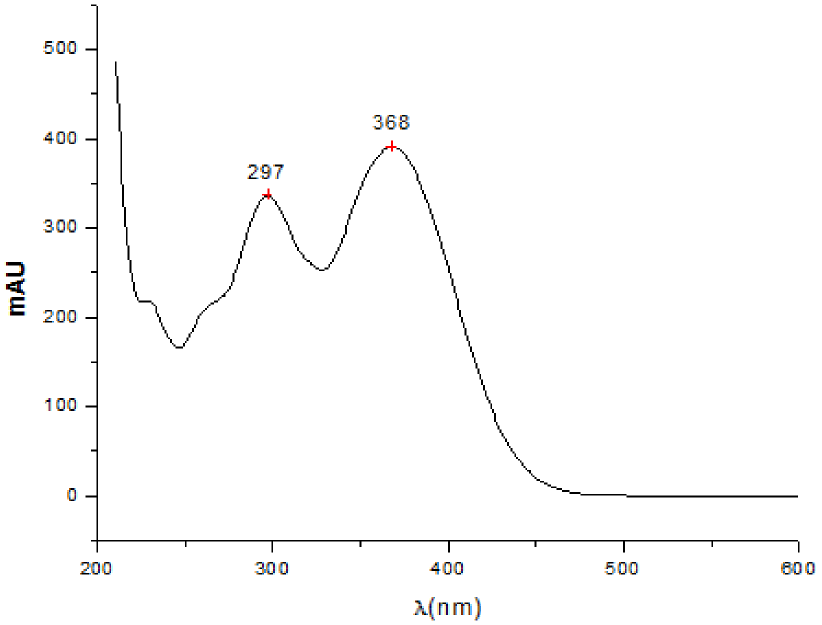

Figure S.28. HPLC-DAD chromatogram of chalcone **9**, MeOH:H_2O (3:1)

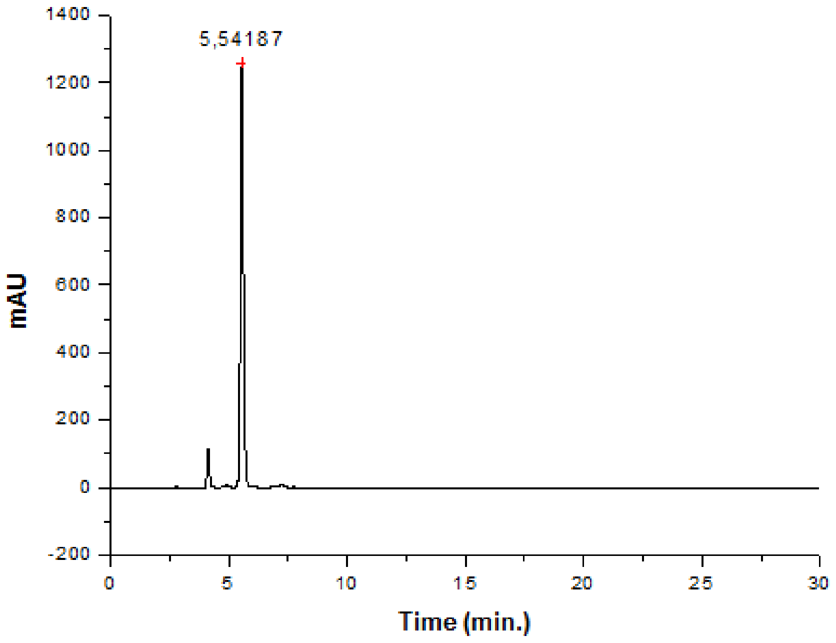

Figure S.29. ^{1}H NMR spectrum (DMSO-d_6, 600 MHz) of chalcone **10**

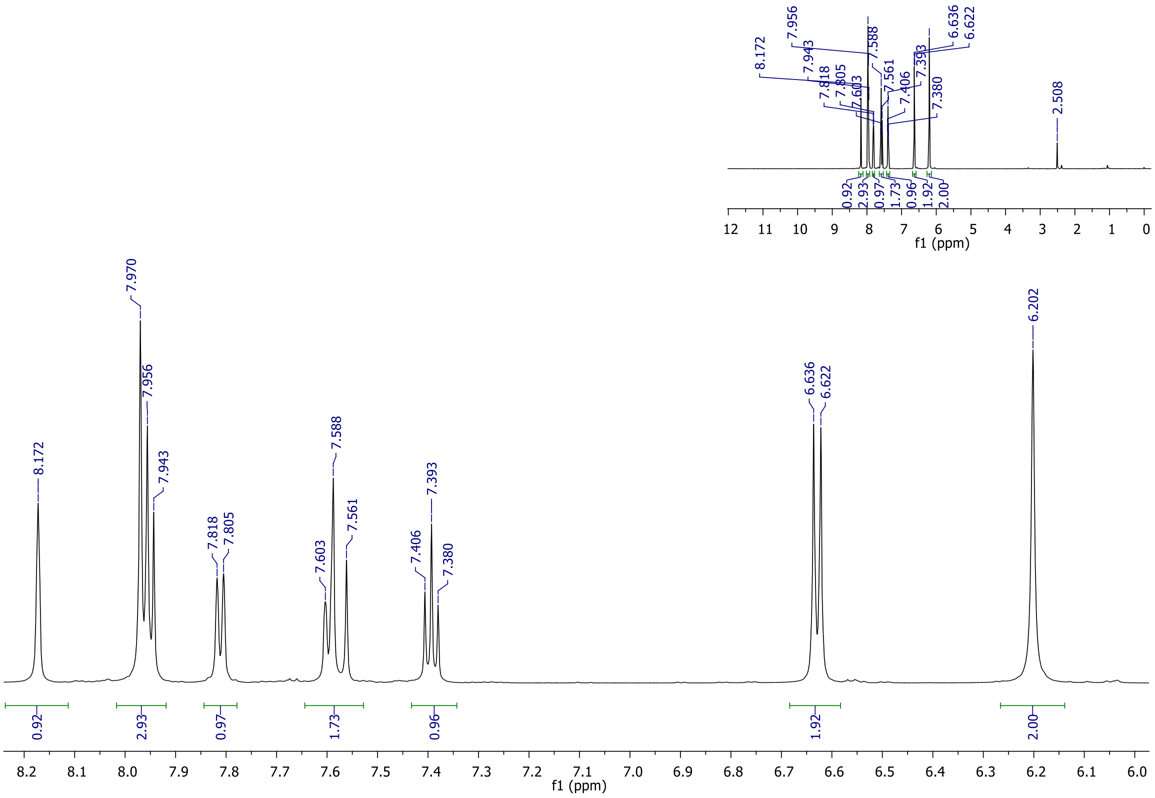

Figure S.30. ^{13}C NMR spectrum (DMSO-d_6, 150 MHz) of chalcone **10**

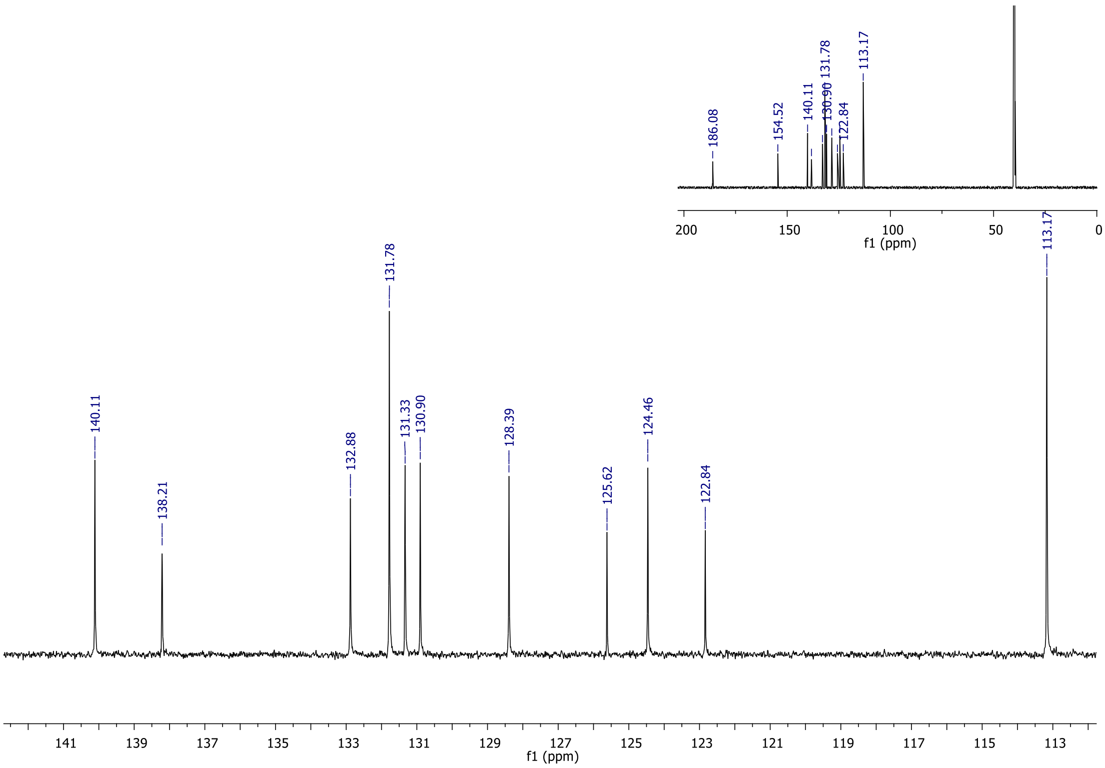

Figure S.31. UV-vis spectrum of chalcone **10** from HPLC-DAD experiment

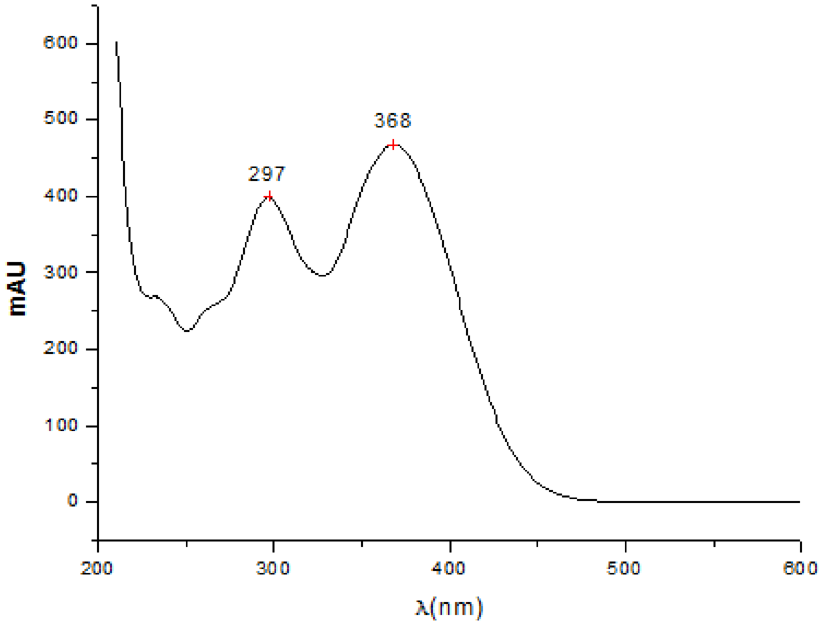

Figure S.32. HPLC-DAD chromatogram of chalcone **10**, MeOH:H_2O (3:1)

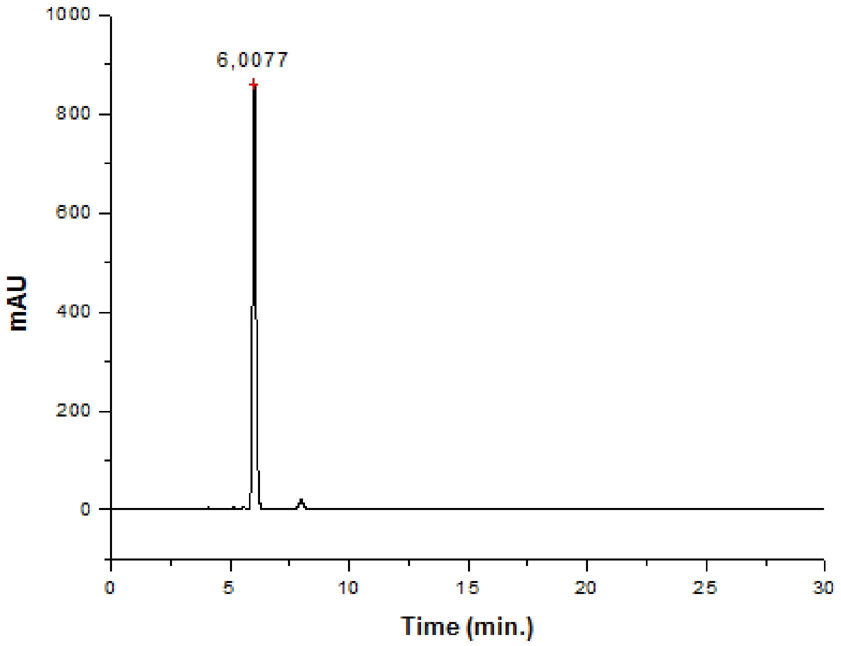

Figure S.33. ^{1}H NMR spectrum (DMSO-d_6, 600 MHz) of chalcone **11**

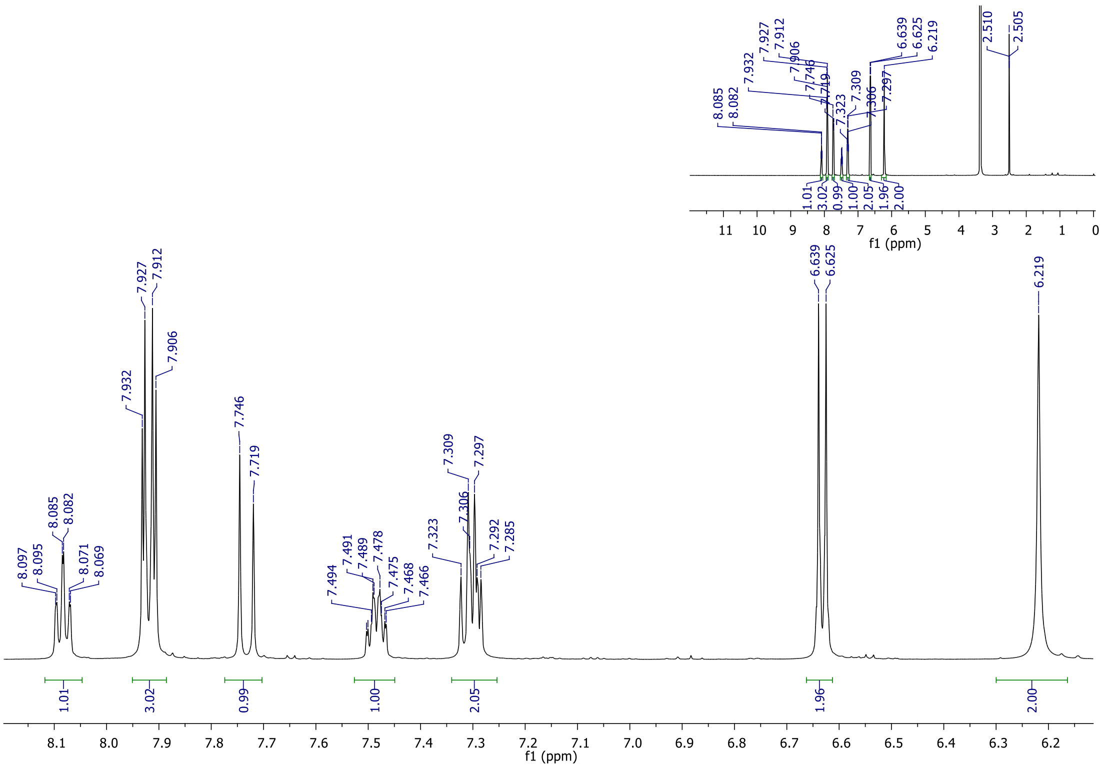

Figure S.34. ^{13}C NMR spectrum (DMSO-d_6, 150 MHz) of chalcone **11**

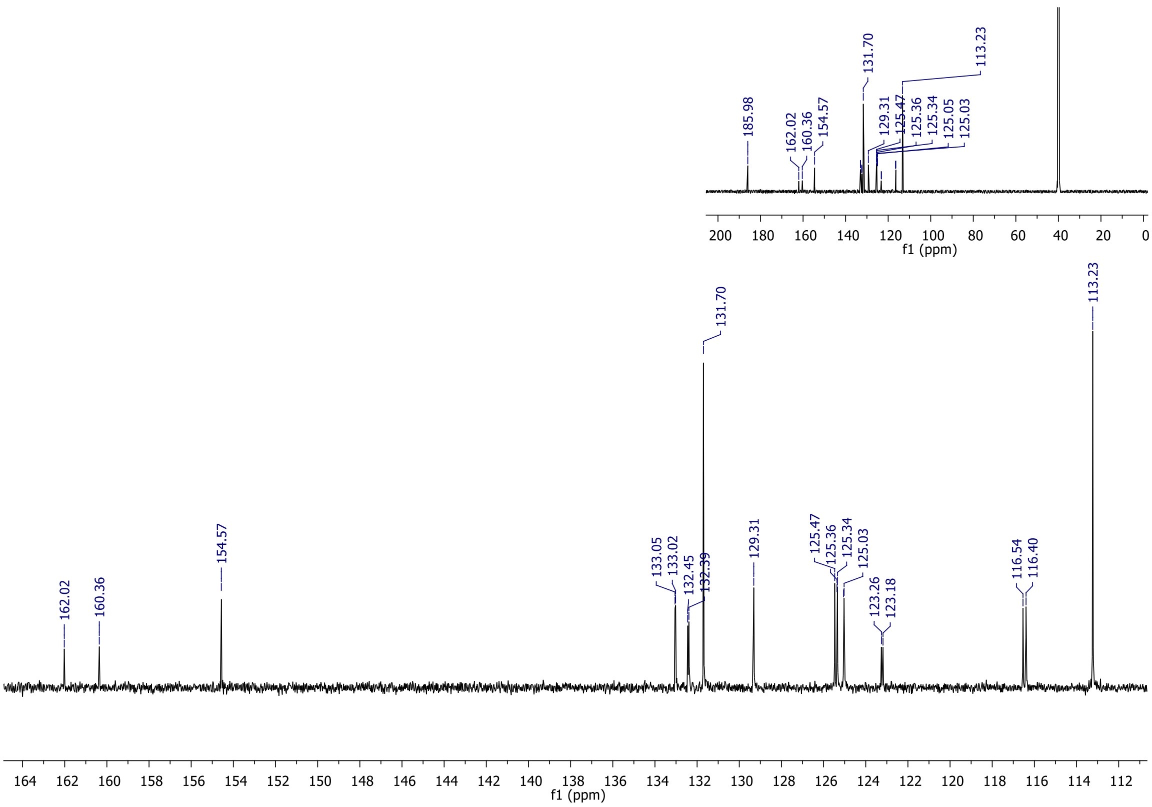

Figure S.35. UV-vis spectrum of chalcone **11** from HPLC-DAD experiment

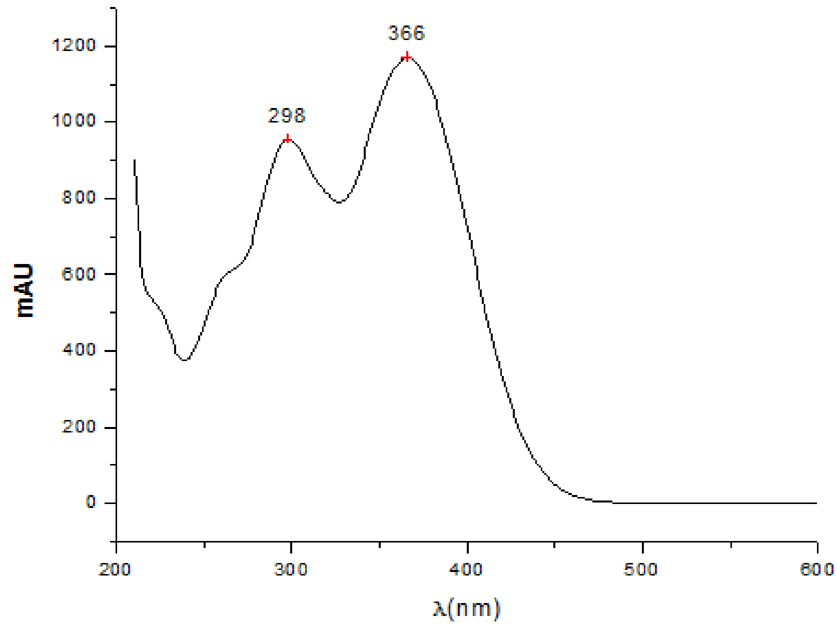

Figure S.36. HPLC-DAD chromatogram of chalcone **11**, MeOH:H_2O (3:1)

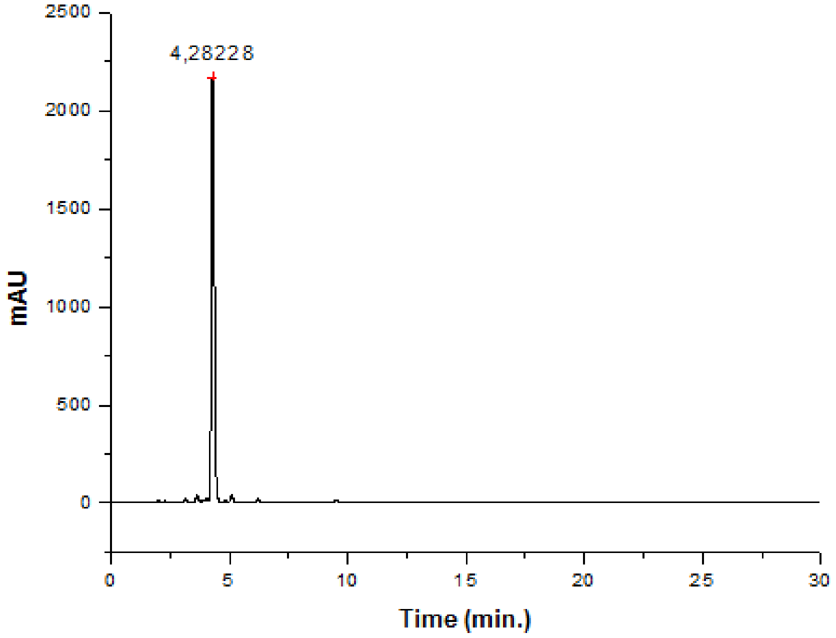

Figure S.37. ^{1}H NMR spectrum (DMSO-d_6, 600 MHz) of chalcone **12**

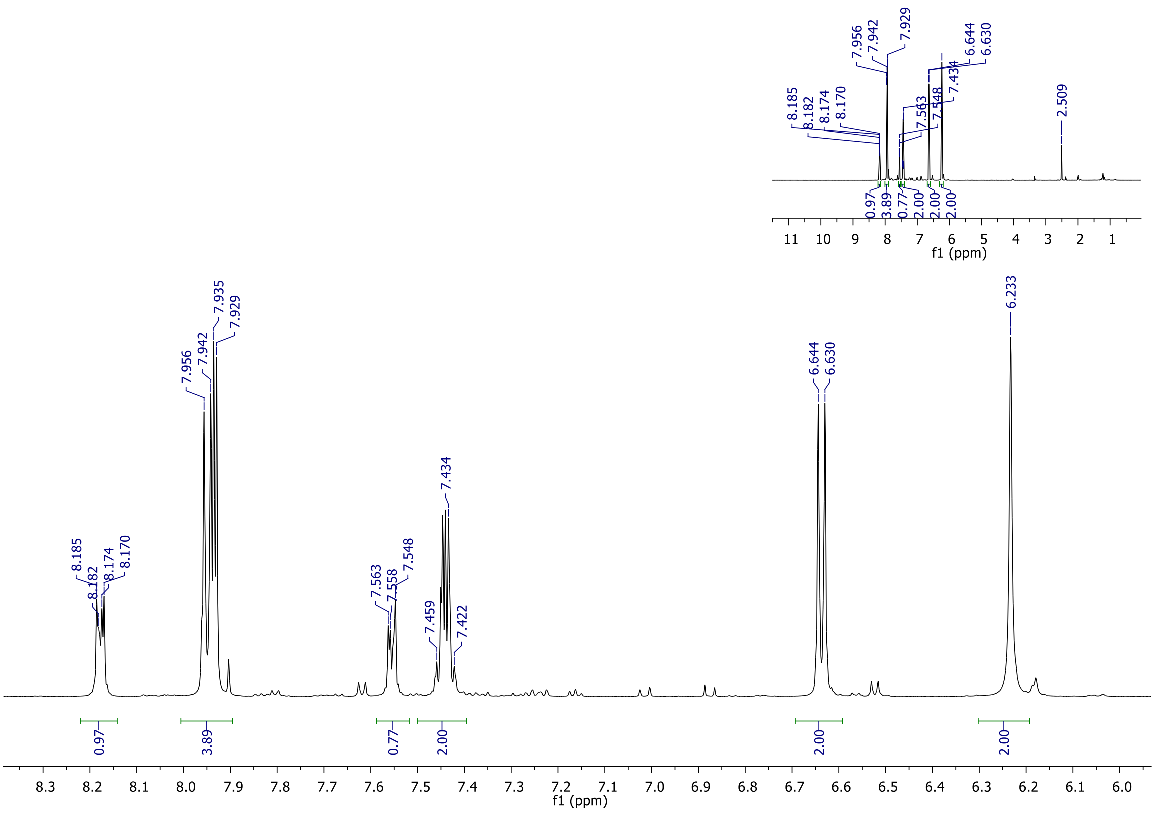

Figure S.38. ^{13}C NMR spectrum (DMSO-d_6, 150 MHz) of chalcone **12**

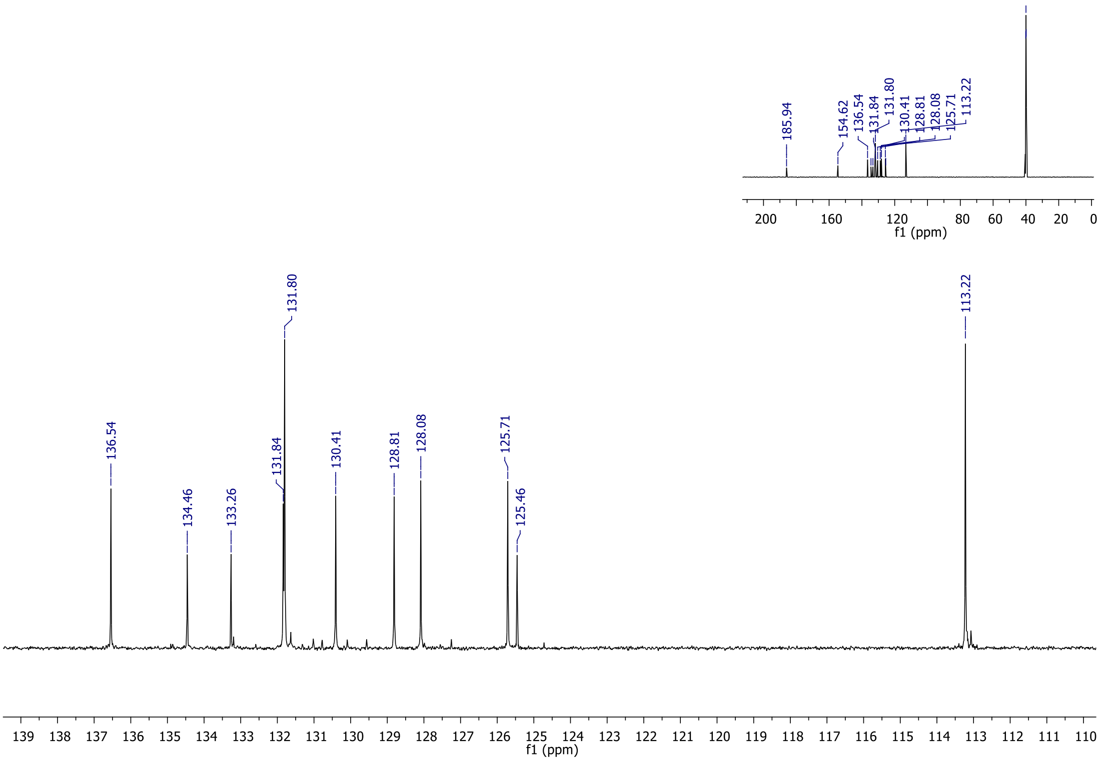

Figure S.39. UV-vis spectrum of chalcone **12** from HPLC-DAD experiment

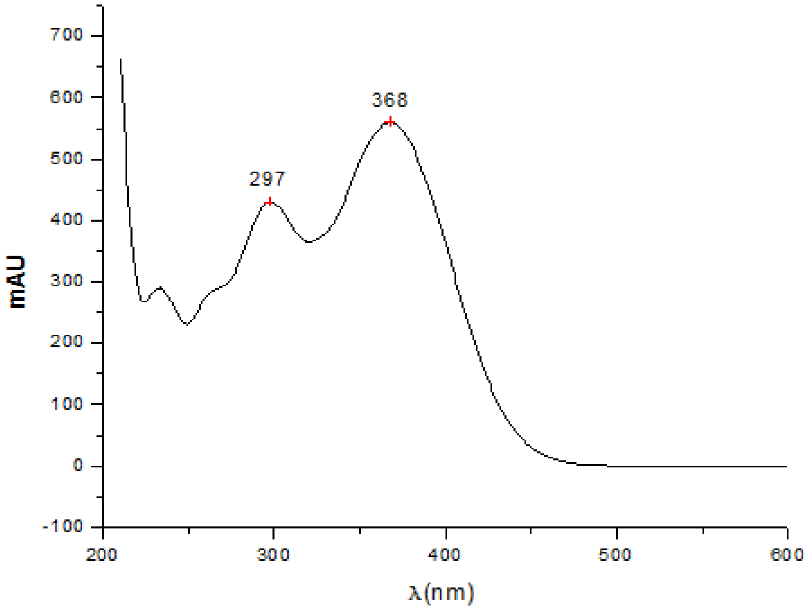

Figure S.40. HPLC-DAD chromatogram of chalcone **12**, MeOH:H_2O (3:1)

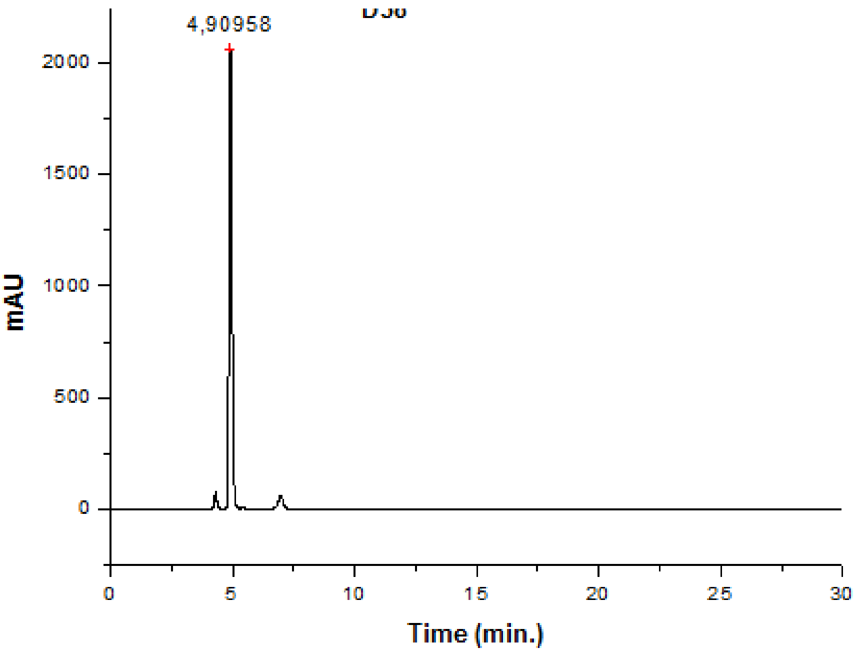

Figure S.41. UV-vis spectrum of chalcone **13** from HPLC-DAD experiment

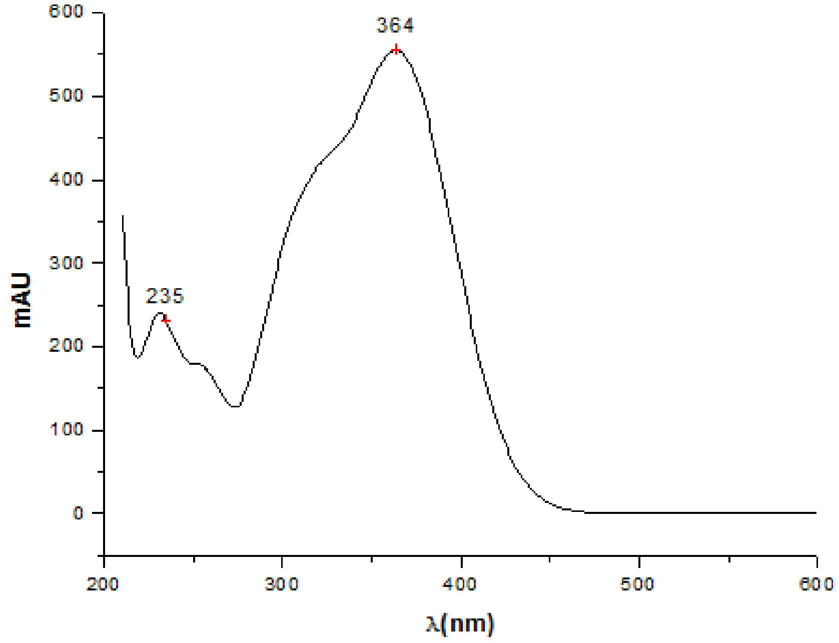

Figure S.42. HPLC-DAD chromatogram of chalcone **13**, MeOH:H_2O (3:1)

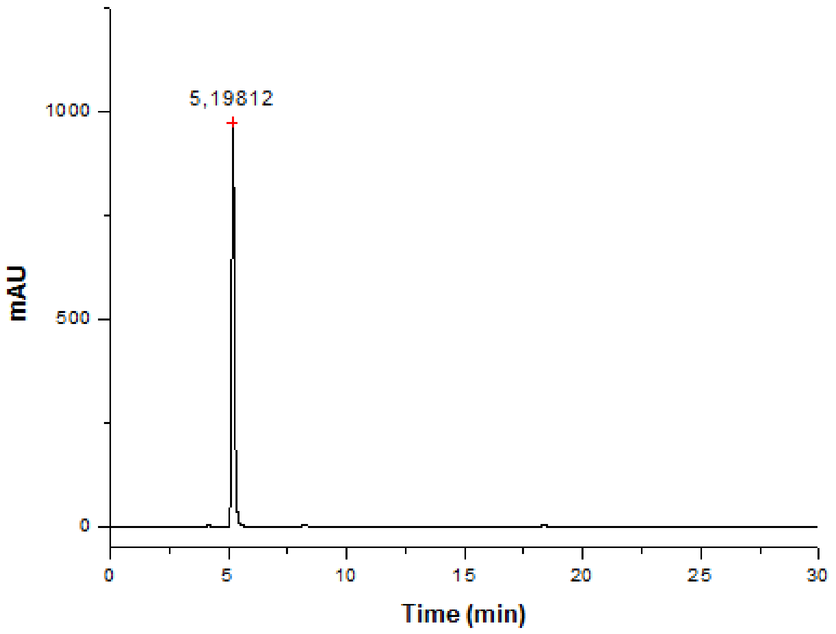

Figure S.43. UV-vis spectrum of chalcone **14** from HPLC-DAD experiment

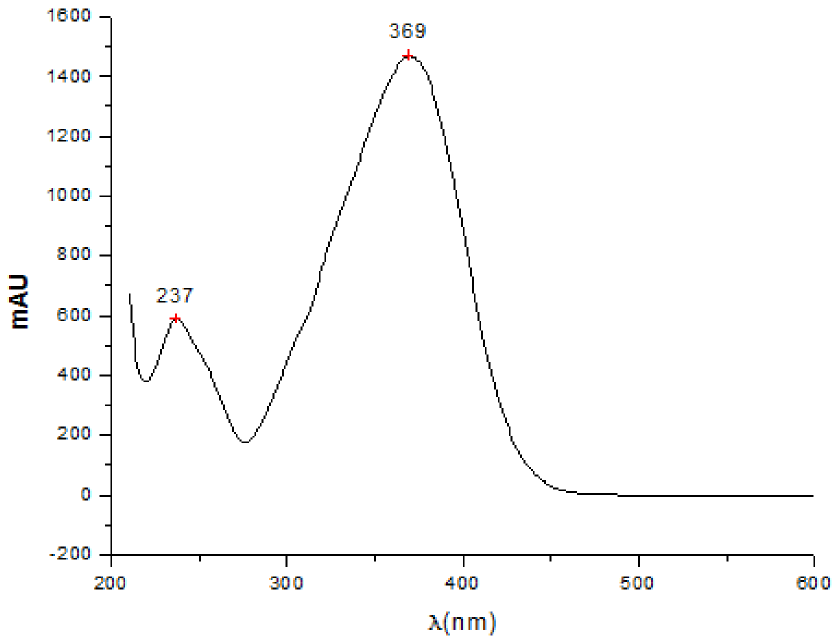

Figure S.44. HPLC-DAD chromatogram of chalcone **14**, MeOH:H_2O (3:1)

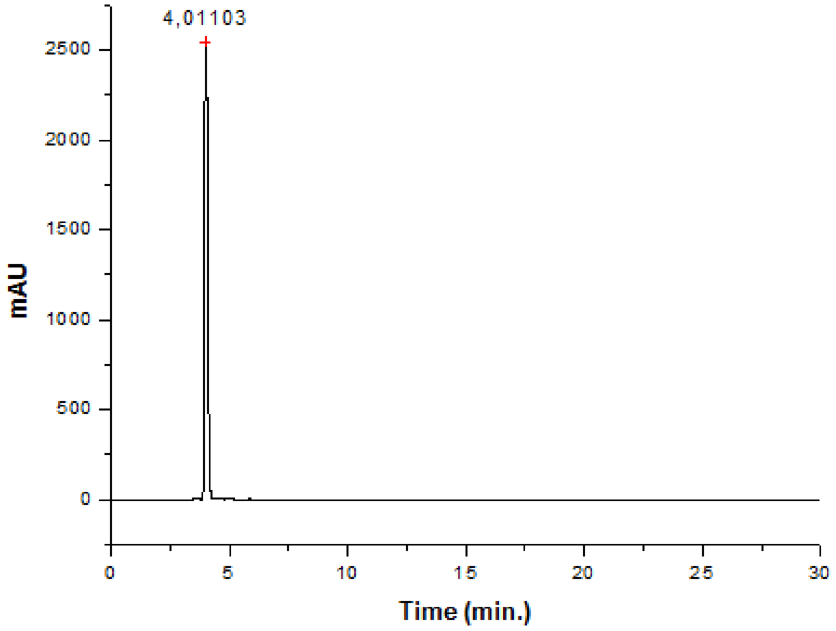

Figure S.45. UV-vis spectrum of chalcone **15** from HPLC-DAD experiment

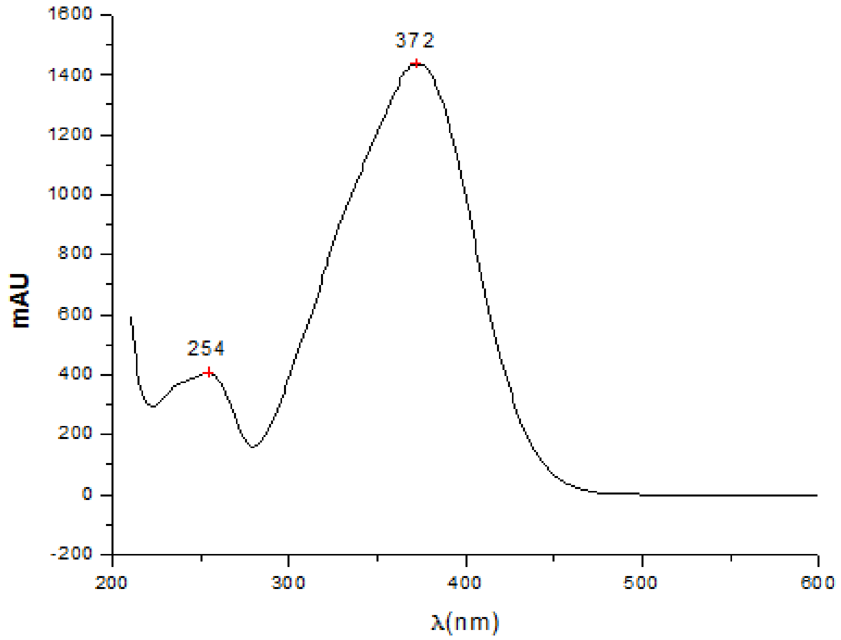

Figure S.46. HPLC-DAD chromatogram of chalcone **15**, MeOH:H_2O (3:1)

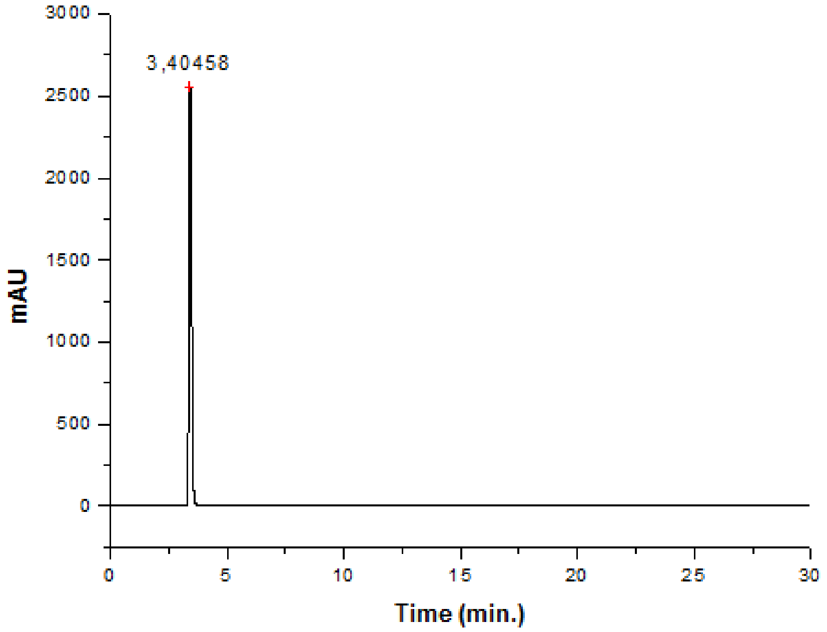

Figure S.47. UV-vis spectrum of chalcone **16** from HPLC-DAD experiment

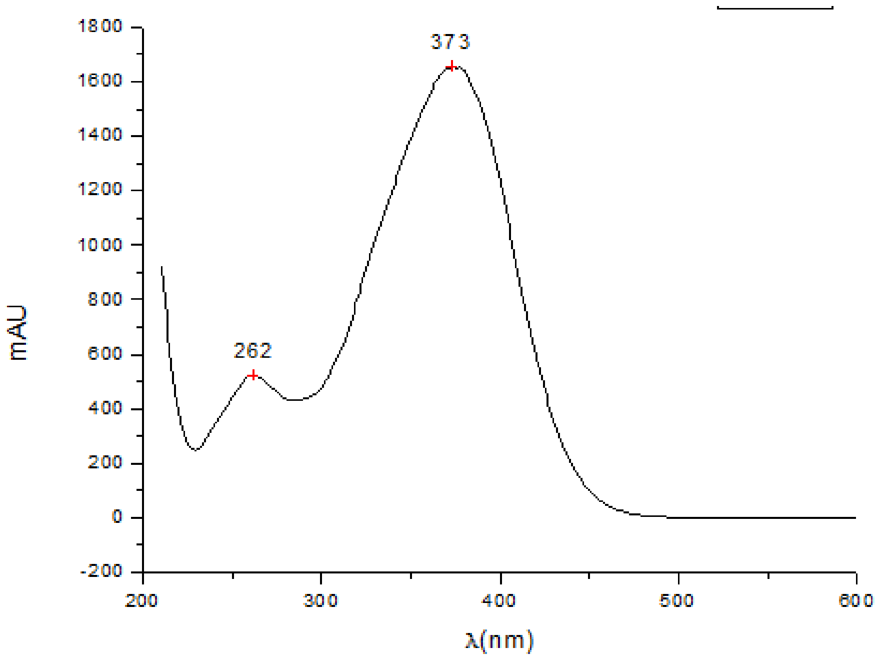

Figure S.48. HPLC-DAD chromatogram of chalcone **16**, MeOH:H_2O (3:1)

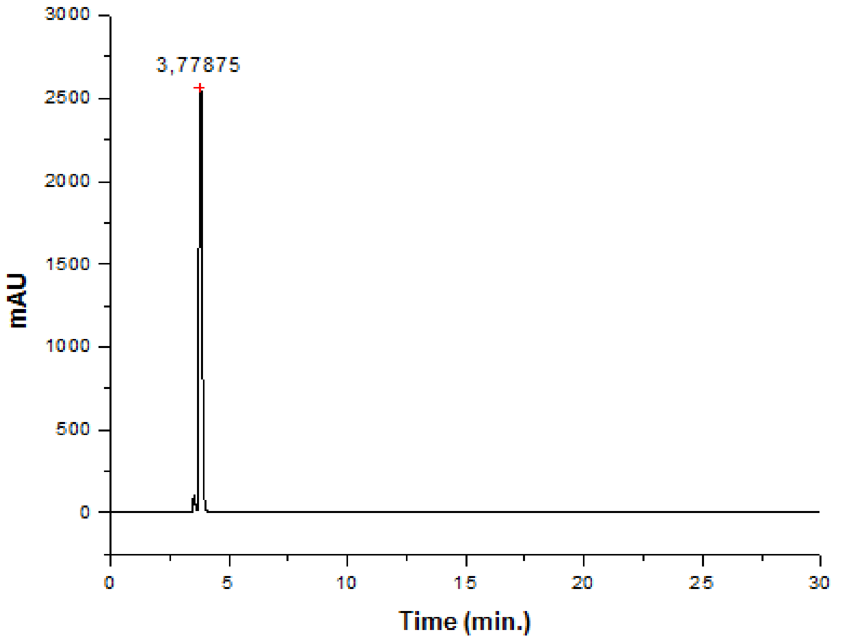

Figure S.49. ^{1}H NMR spectrum (DMSO-d_6, 600 MHz) of chalcone **17**

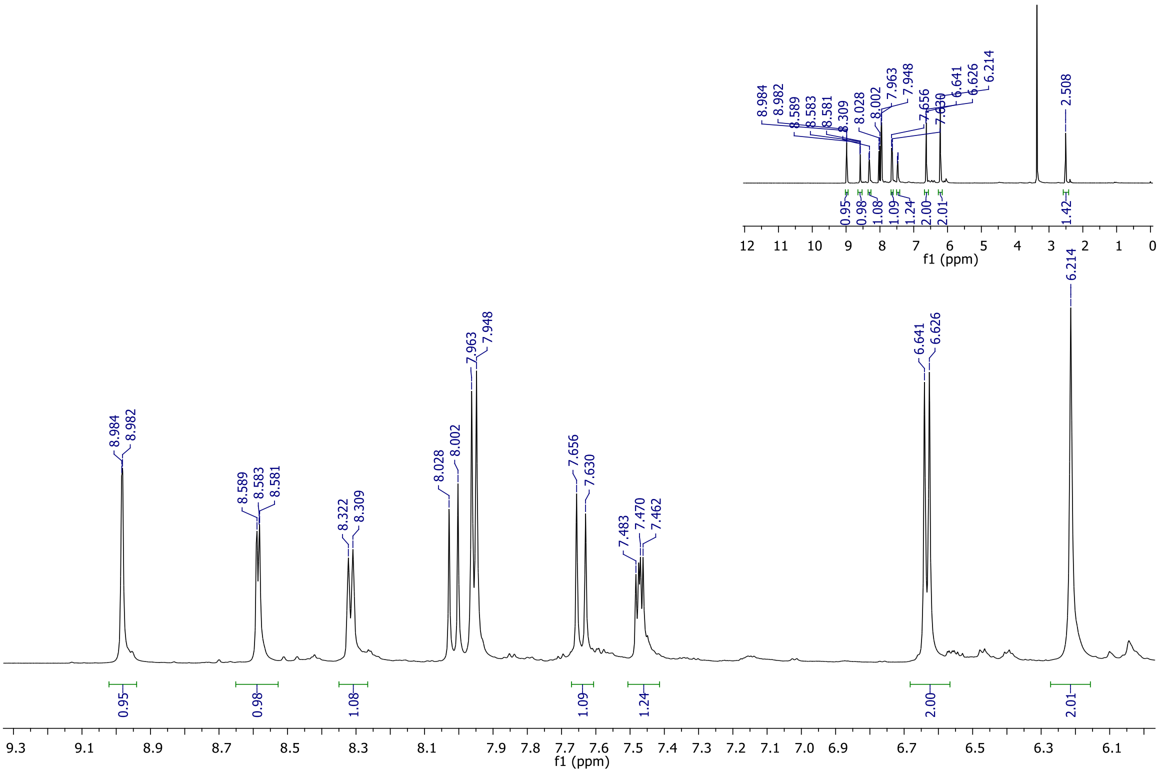

Figure S.50. ^{13}C NMR spectrum (DMSO-d_6, 150 MHz) of chalcone **17**

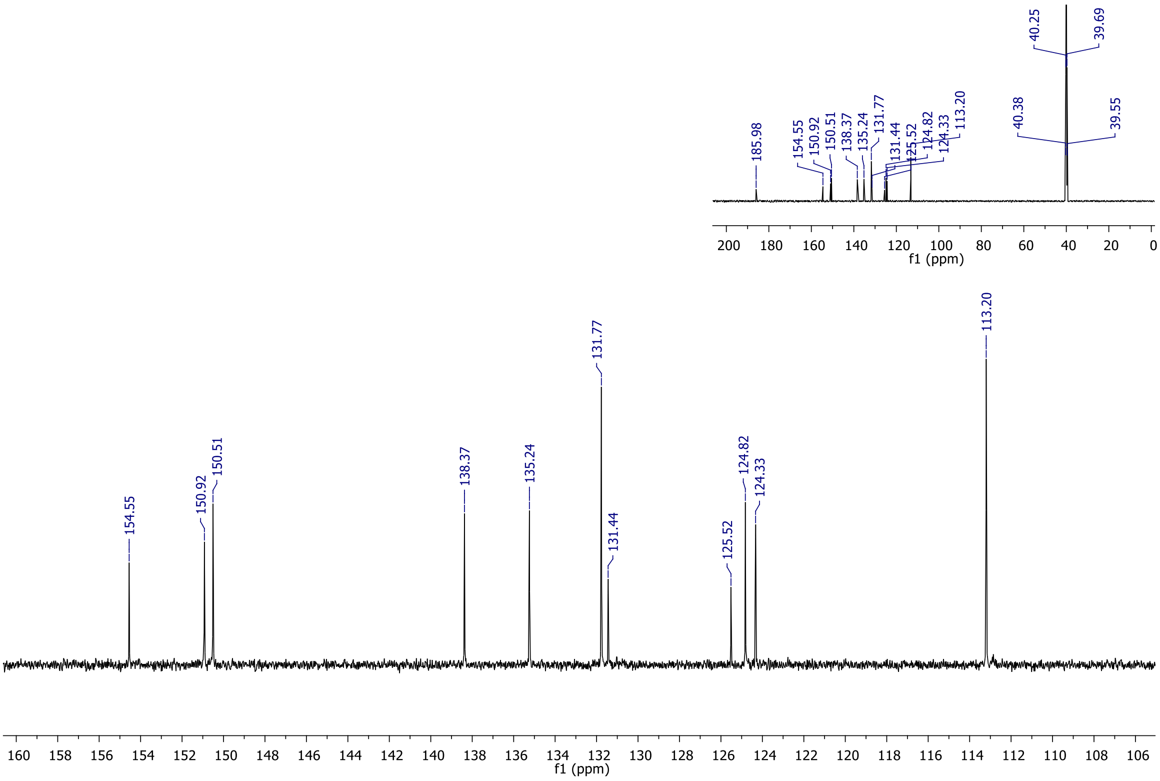

Figure S.51. UV-vis spectrum of chalcone **17** from HPLC-DAD experiment

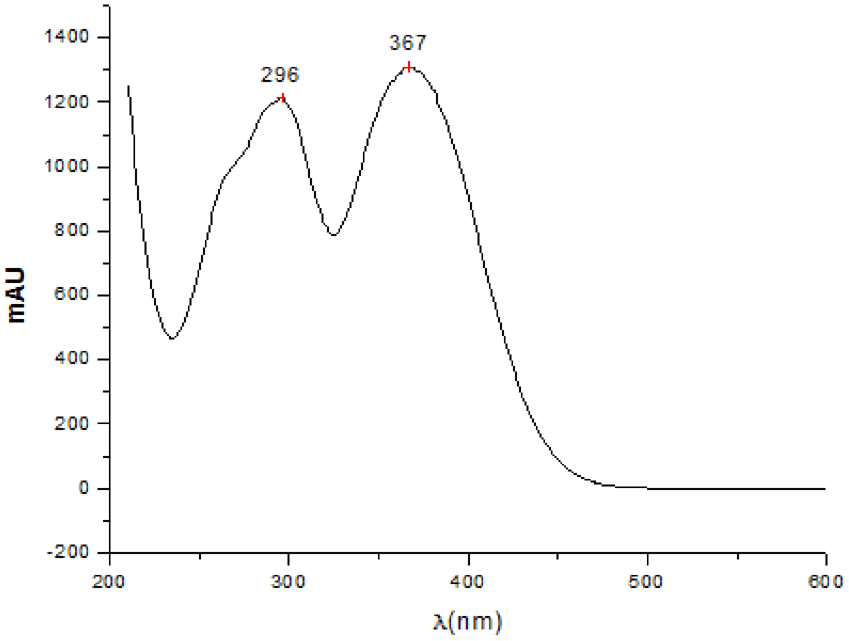

Figure S.52. HPLC-DAD chromatogram of chalcone **17**, MeOH:H_2O (3:1)

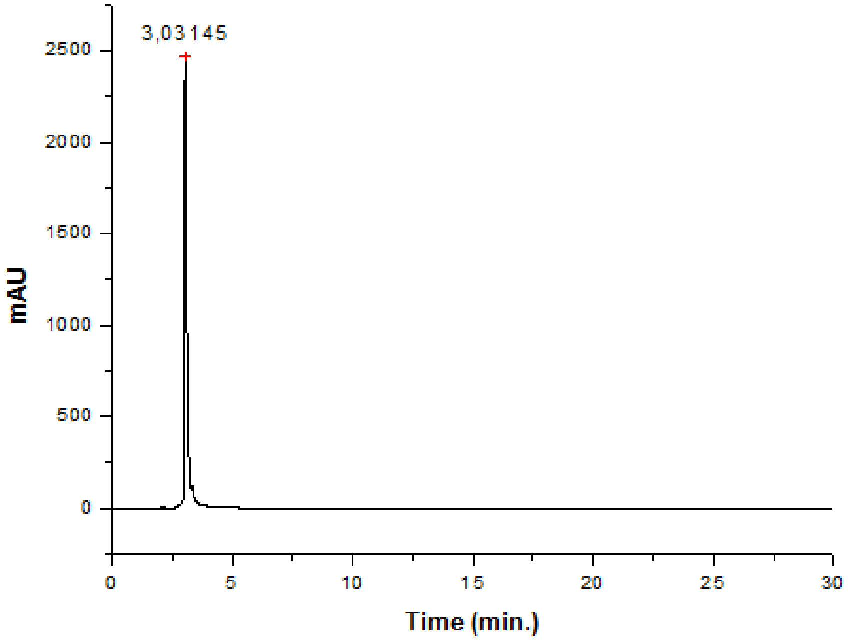

Figure S.53. ^{1}H NMR spectrum (DMSO-d_6, 600 MHz) of chalcone **18**

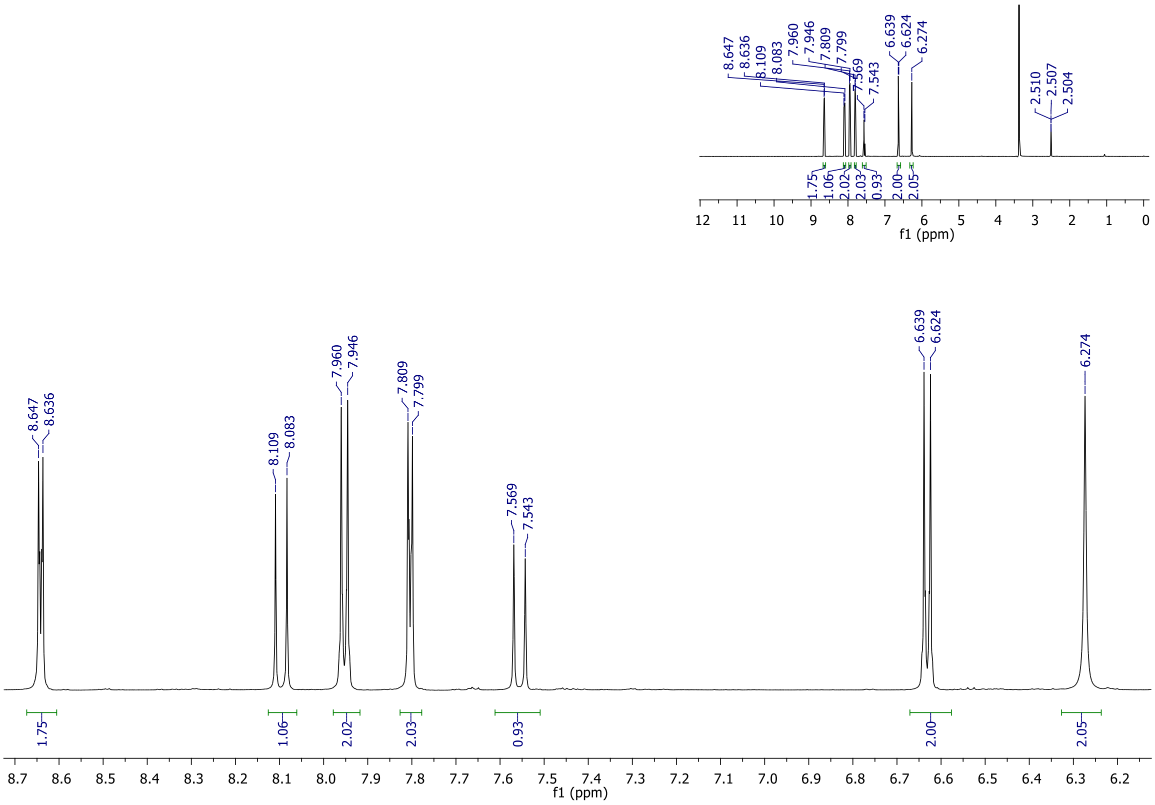

Figure S.54. ^{13}C NMR spectrum (DMSO-d_6, 150 MHz) of chalcone **18**

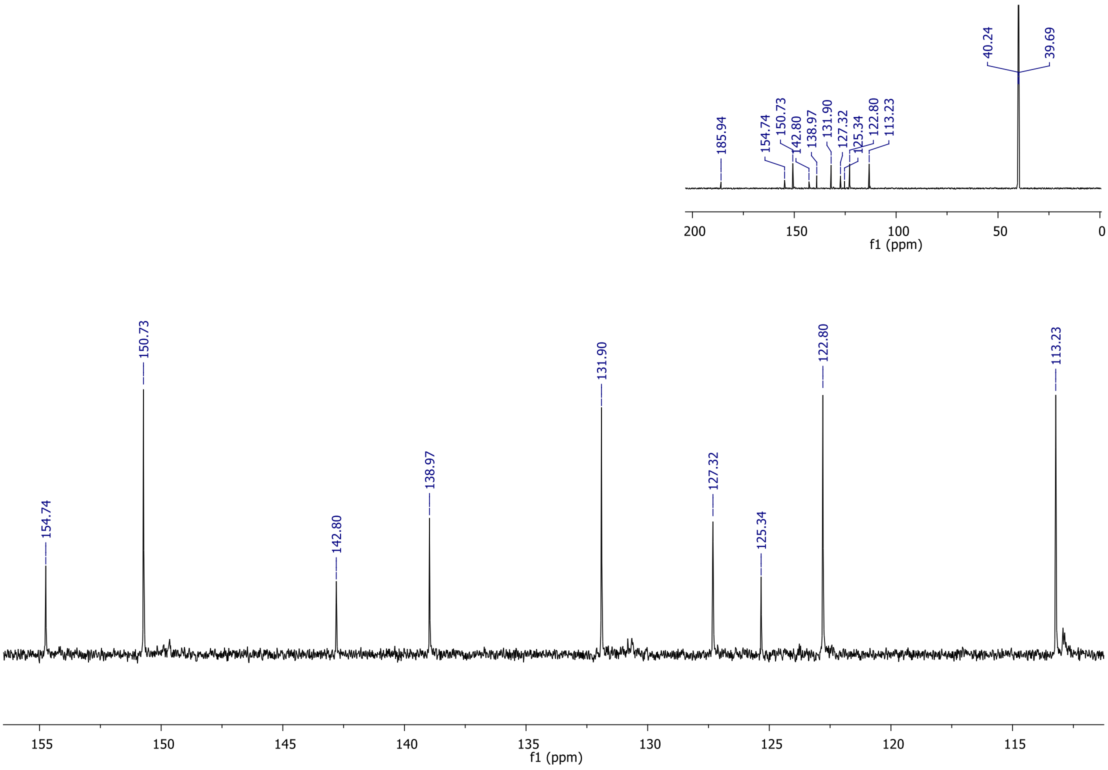

Figure S.55. UV-vis spectrum of chalcone **18** from HPLC-DAD experiment

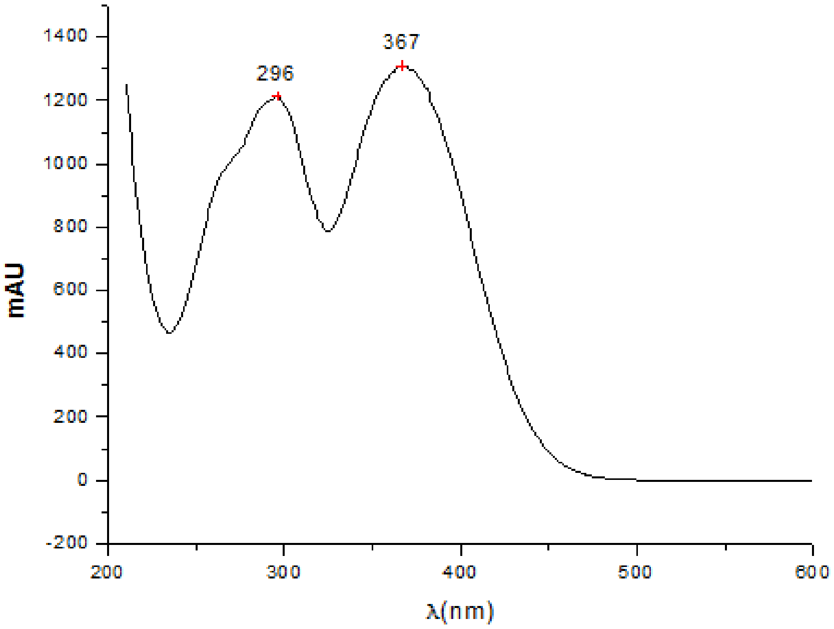

Figure S.56. HPLC-DAD chromatogram of chalcone **18**, MeOH:H_2O (3:1)

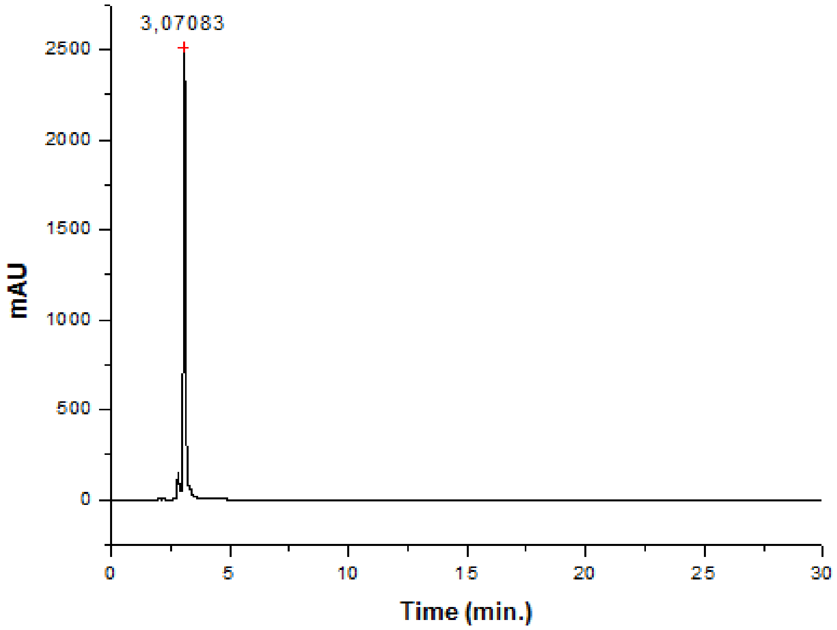

Figure S.57. UV-vis spectrum of chalcone **19** from HPLC-DAD experiment

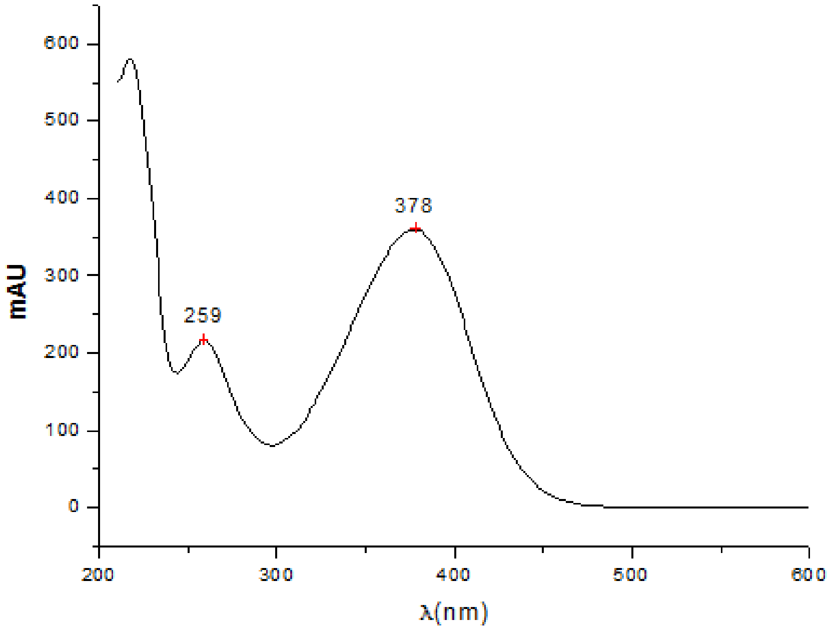

Figure S.58. HPLC-DAD chromatogram of chalcone **19**, MeOH:H_2O (3:1)

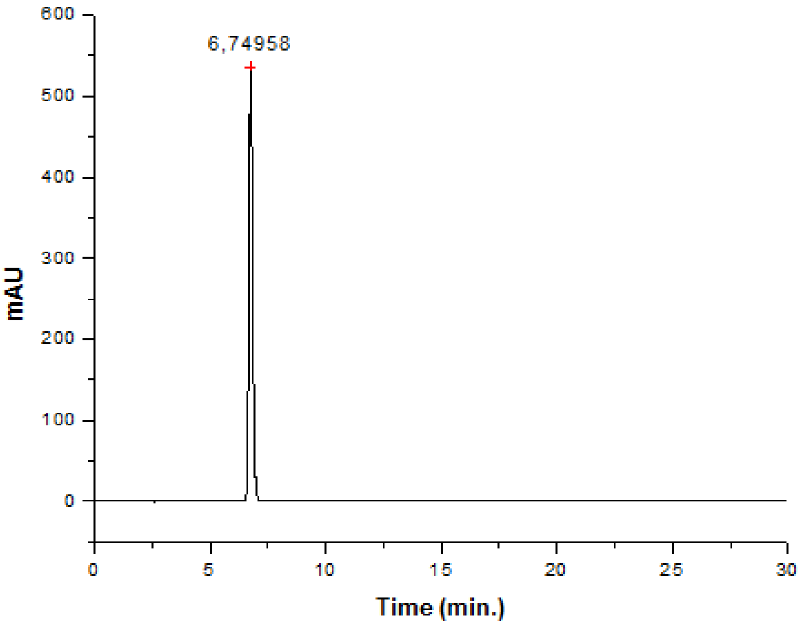

Figure S.59. ^{1}H NMR spectrum (DMSO-d_6, 600 MHz) of chalcone **20**

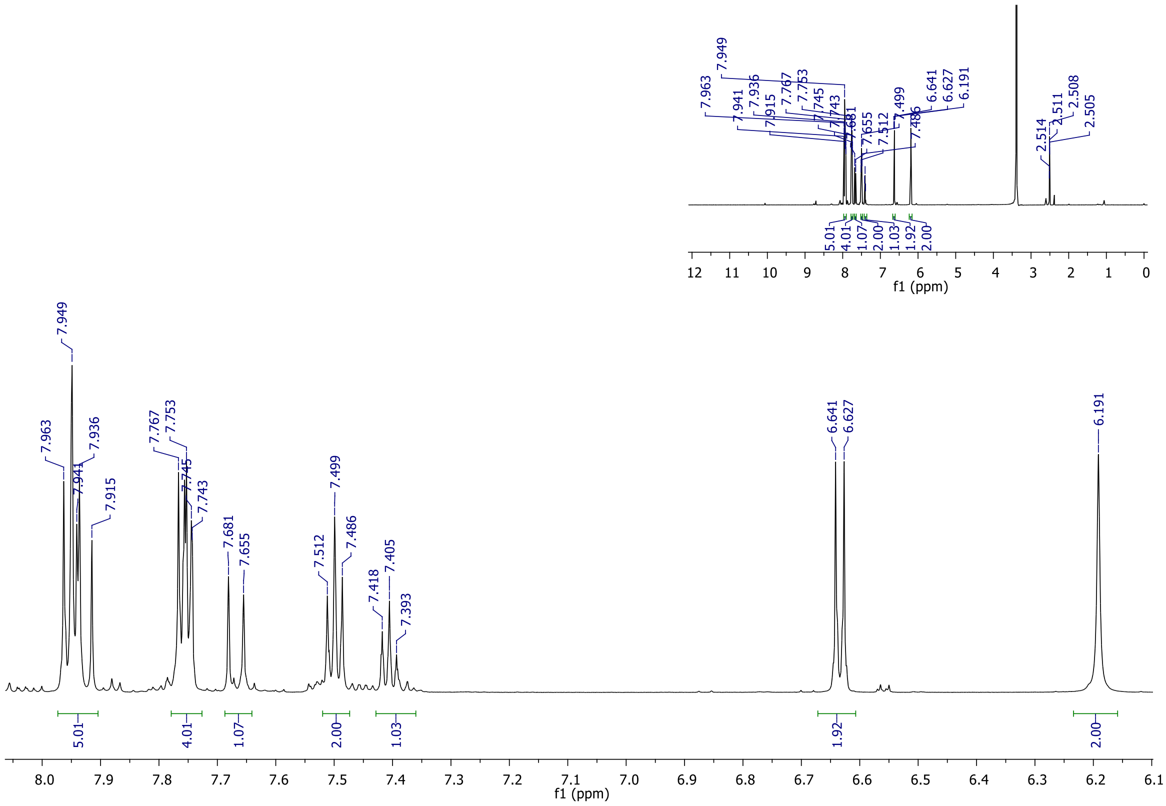

Figure S.60. ^{13}C NMR spectrum (DMSO-d_6, 150 MHz) of chalcone **20**

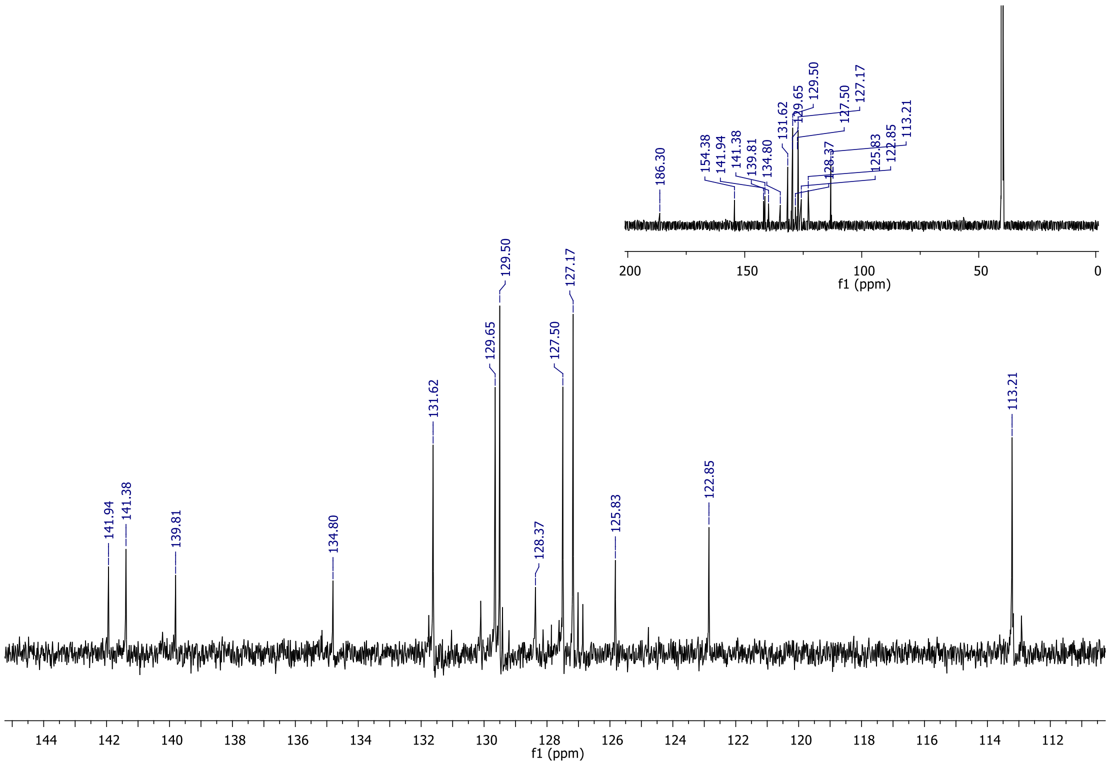

Figure S.61. UV-vis spectrum of chalcone **20** from HPLC-DAD experiment

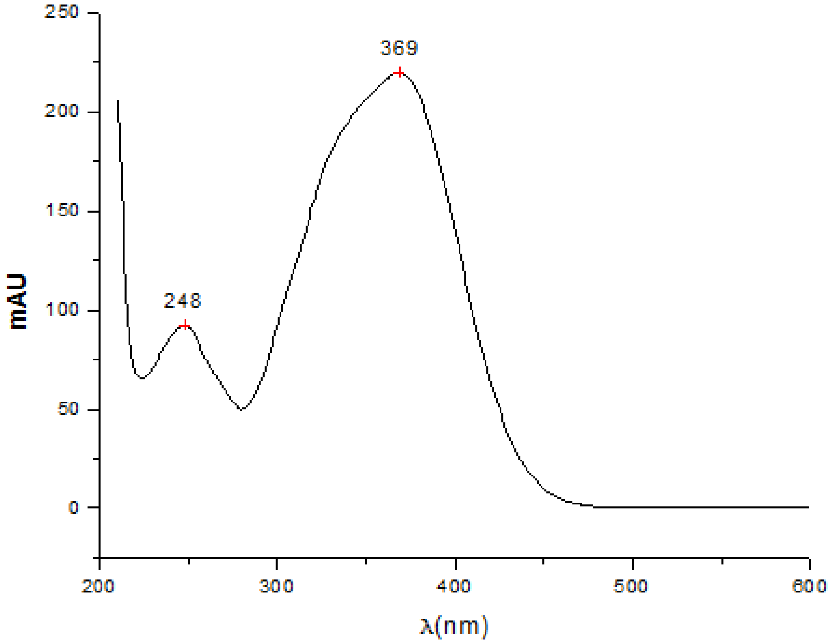

Figure S.62. HPLC-DAD chromatogram of chalcone **20**, MeOH:H_2O (3:1)

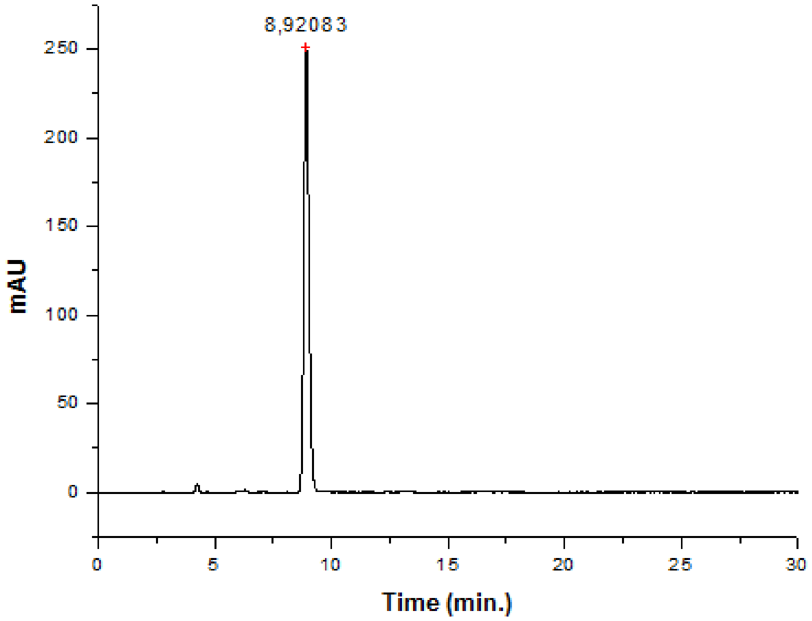

References

1. Santos MB, Pinhanelli VC, Garcia MAR, Silva G, Baek SJ, França SC, Fachin AL, Marins M, Regasini LO. Antiproliferative and pro-apoptotic activities of 2′- and 4′-aminochalcones against tumor canine cells. Eur J M Chem. 2017;138:884–889.

2. Nazir S, Ansari FL, Hussain T, Mazhar K, Muazzam AG, Qasmi Z-H, Makhmoor T, Noureen H, Mirza B. Brine shrimp lethality assay 'an effective prescreen': Microwave-assisted synthesis, BSL toxicity and 3DQSAR studies-based designing, docking and antitumor evaluation of potent chalcones. Pharmaceutical Biology. 2013;51(9):1091–1103.

3. Jardim GAM, Guimarães TT, Pinto M do CFR, Cavalcanti BC, de Farias KM, Pessoa C, Gatto CC, Nair DK, Namboothiri INN, da Silva Júnior EN. Naphthoquinone-based chalcone hybrids and derivatives: synthesis and potent activity against cancer cell lines. MedChemComm. 2015;6(1):120–130.

4. Chintakrindi AS, Gohil DJ, Kothari ST, Chowdhary AS, Kanyalkar MA. Design, synthesis and evaluation of chalcones as H1N1 Neuraminidase inhibitors. Medicinal Chemistry Research. 2018;27(4):1013–1025.

5. Thirumurthy K, Thirunarayanan G. A facile designed highly moderate craspedia flowerlike sulphated Bi 2 O 3 -fly ash catalyst: Green synthetic strategy for (6 H -pyrido[3,2-b]carbazol-4-yl)aniline derivatives in water. Arabian Journal of Chemistry. 2018;11(4):443–452.

6. Blickenstaff RT, Hanson WR, Reddy S, Witt R. Potential radioprotective agents--VI. Chalcones, benzophenones, acid hydrazides, nitro amines and chloro compounds. Radioprotection of murine intestinal stem cells. Bioorganic & Medicinal Chemistry. 1995;3(7):917–922.

7. Yi F, Peng Y, Song G, Li J. Solid phase synthesis of aminochalcones. Journal of Chemical Research. 2005;2005(5):311–312.

8. Chu W-C, Bai P-Y, Yang Z-Q, Cui D-Y, Hua Y-G, Yang Y, Yang Q-Q, Zhang E, Qin S. Synthesis and antibacterial evaluation of novel cationic chalcone derivatives possessing broad spectrum antibacterial activity. European Journal of Medicinal Chemistry. 2018;143:905–921.

9. Mai CW, Yaeghoobi M, Abd-Rahman N, Kang YB, Pichika MR. Chalcones with electron-withdrawing and electron-donating substituents: Anticancer activity against TRAIL resistant cancer cells, structure–activity relationship analysis and regulation of apoptotic proteins. Eur J Med Chem. 2014;77:378–387.

10. Selvam P, Babu KV, Nanjundan S. Synthesis, characterization and photocrosslinking properties of polyacrylamides having bromo substituted pendant cinnamoyl moieties. European Polymer Journal. 2005;41(1):35–45.

11. Gan X, Wang Y, Hu D, Song B. Design, Synthesis, and Antiviral Activity of Novel Chalcone Derivatives Containing a Purine Moiety. Chinese Journal of Chemistry. 2017;35(5):665–672.

12. Prasad YR, Rani VJ, Rao AS. In vitro Antioxidant Activity and Scavenging Effects of Some Synthesized 4'-Aminochalcones. Asian Journal of Chemistry. 2013;25(1):52–58.

13. Amir M, Ali S, K S. Synthesis and biological evaluation of some new pyrazoline derivative as anti-inflammatory agents. Indian Journal of Chemistry. 2016;55B:478–485.

14. Atla SR, Sistla R, Yejella RP. Study of the Ant-Tumor Activity of Synthetic Pyrido [2,3-*d*] Pyrimidines, Pyrimidines and Corresponding Chalcone Derivatives. 2018;9(9):3804–3814.
